# Supplementary material for: Photocrosslinking Activity-Based Probes for Ubiquitin RING E3 Ligases
Source: Cell Chem Biol. 2020 Jan 16;27(1):74–82.e6. doi: 10.1016/j.chembiol.2019.11.013 (PMC6963778; doi:10.1016/j.chembiol.2019.11.013)
Supplement: Document S1. Figures S1–S4 and Table S1 [file mmc1.pdf]

Cell Chemical Biology, Volume *27*

Supplemental Information

Photocrosslinking Activity-Based Probes for Ubiquitin RING E3 Ligases

Sunil Mathur, Adam J. Fletcher, Emma Branigan, Ronald T. Hay, and Satpal Virdee

Photocrosslinking Activity-Based Probes for Ubiquitin RING E3 Ligases

Supplemental Information

Sunil Mathur[a], Adam J. Fletcher[a], Emma Branigan[b], Ronald T. Hay[b] and Satpal Virdee*[a]

[a] Dr. S. Mathur, Dr. A.J. Fletcher, Dr S. Virdee
MRC Protein Phosphorylation and Ubiquitylation Unit
University of Dundee
Scotland, UK
E-mail: s.s.virdee@dundee.ac.uk

[b] Dr. E. Branigan, Prof. R.T. Hay
Division of Gene Regulation and Expression
University of Dundee
Scotland, UK

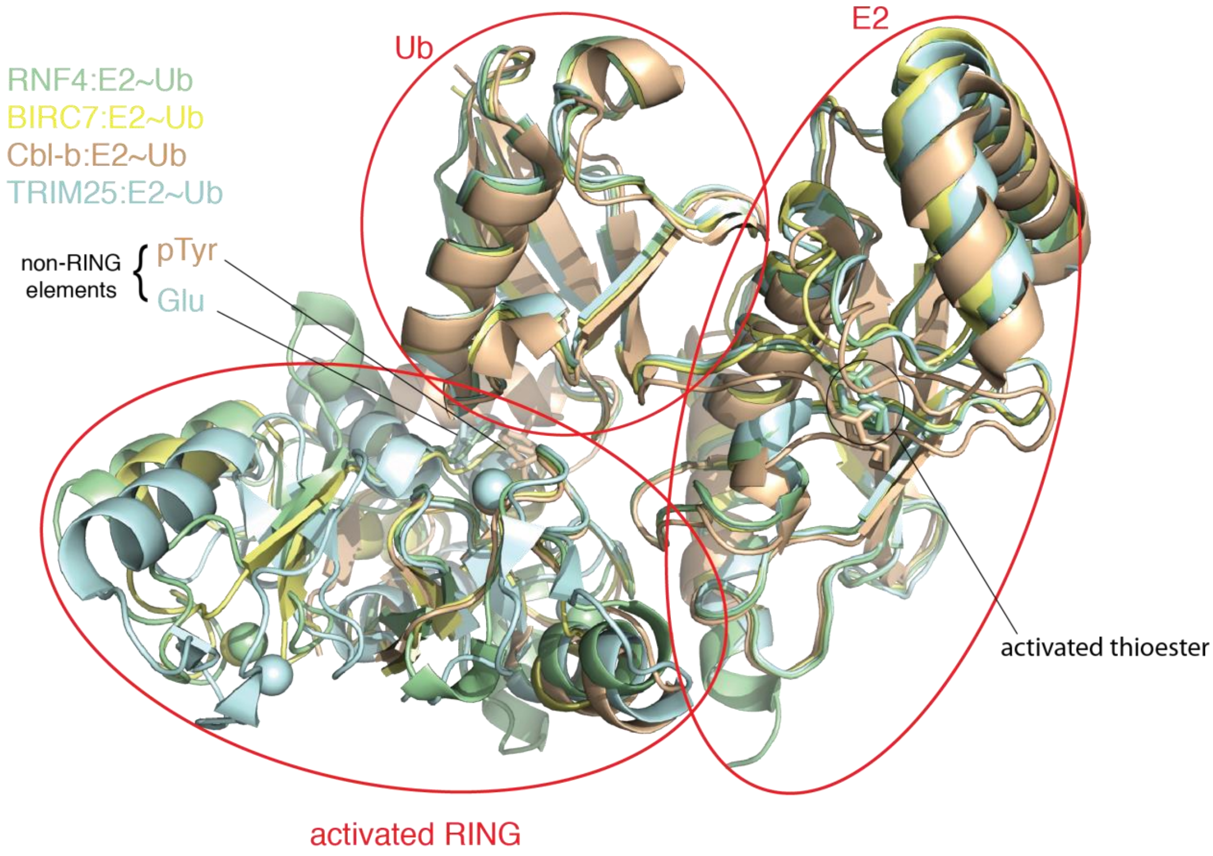

Figure S1. Structural superposition of activated RING:E2~Ub complexes, related to Figure 1. Activated RING E3s bind E2~Ub and induce a "closed" E2~Ub conformation which activates the thioester for aminolysis (NB, in the presented crystal structures the labile thioester has been replaced with an ester or isopeptide linkage by mutation of the E2 catalytic cysteine to serine or lysine, respectively). The closed conformation is induced by binding of the RING to a composite E2~Ub interface. Of particular significance, the Ub component is held in the closed conformation by interactions with RING protomer 2 in the case of RNF4 and BIRC7. For Cbl-b, a phosphotyrosine moiety (pTyr) serves as a non-RING element and facilitates the closed conformation. In the case of TRIM25, dimerization and the presence of a non-RING glutamate (Glu) residue facilitates the closed conformation.

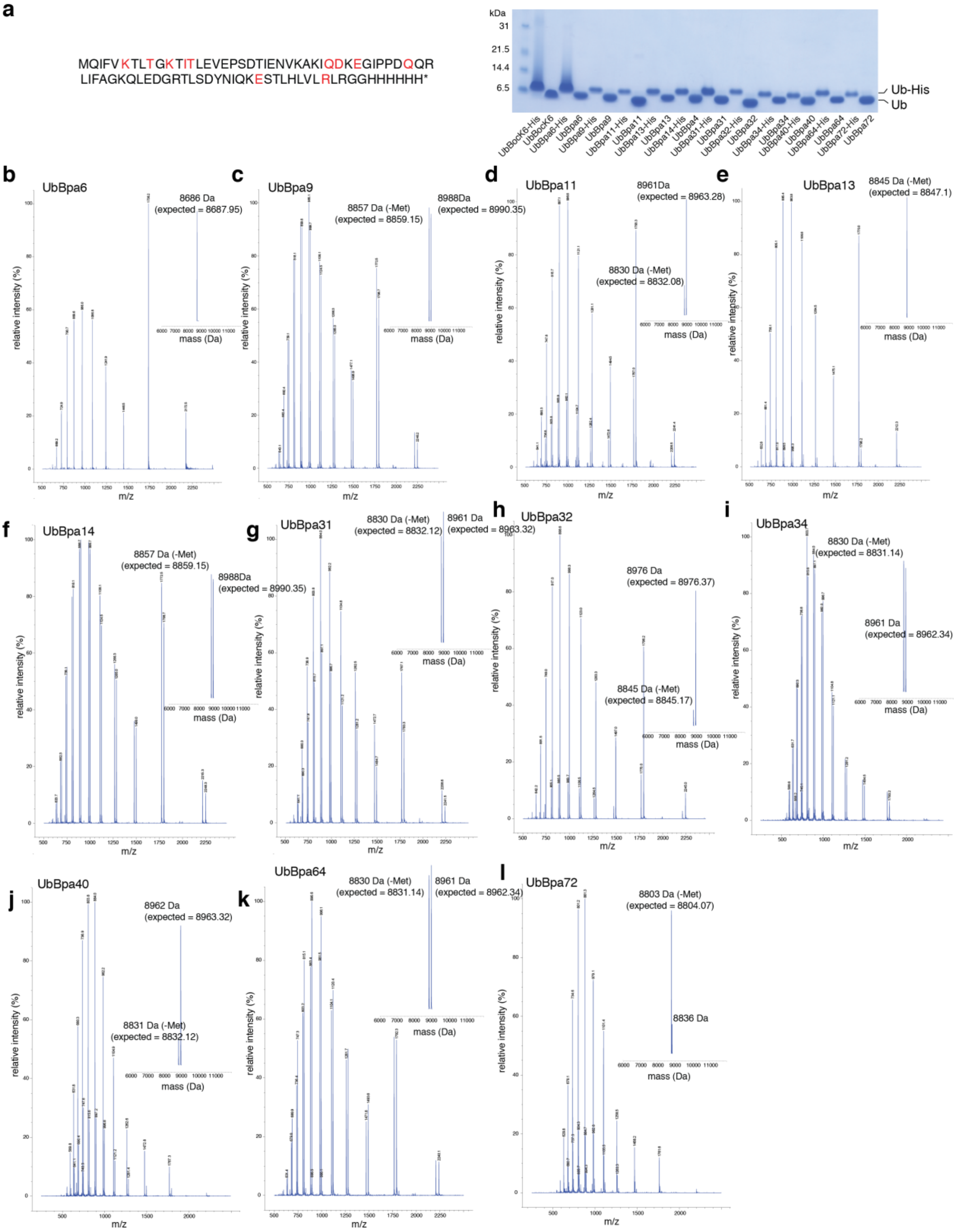

Figure S2. Incorporation of the photocrosslinking amino acid p-benzoyl-L-phenylalanine (Bpa) into RING-proximal sites of ubiquitin, related to Figure 2. a) Ubiquitin sequence with mutated residues highlighted in red and SDS-PAGE analysis and visualization by Coomasie staining. As a positive control, the reference amino acid t-butyloxycarbonyl-L-lysine (BocK) was incorporated into Ub at position 6 (Pao et al., 2016). To facilitate purification a C-terminal His tag was appended to Ub which was subsequently removed by treatment with the deubiquitinating enzyme UCH-L3 (Virdee et al., 2010). For each mutant, samples pre- and post-UCH-L3 treatment were analysed. **b)** Electrospray

ionization mass spectra for ubiquitin Bpa mutants. Spectra correspond to Ub after cleavage of the C-terminal His-tag. With the exception of the amber mutant clone for position 6, Ub is expressed with an N-terminal MGS motif. Introduction of the DNA coding sequence for this facilitated cloning. For these latter clones the N-terminal methionine is cleaved by cellular methionyl aminopeptidase to various degrees. UbBpa6, observed mass = 8686 Da; expected = 8787.95 Da. **c)** UbBpa9 (-Met), observed mass = 8857 Da; expected = 8859.15 Da. UbBpa9, observed mass = 8988 Da; expected = 8990.35 Da. **d)** UbBpa11 (-Met), observed mass = 8830 Da; expected = 8832.08 Da. UbBpa11, observed mass = 8961 Da; expected = 8963.28 Da. **e)** UbBpa13 (-Met), observed mass = 8845 Da; expected = 8847.1 Da. **f)** UbBpa14 (-Met), observed mass = 8857 Da; expected = 8859.15 Da. UbBpa14, observed mass = 8988 Da; expected = 8990.35 Da. **g)** UbBpa31 (-Met), observed mass = 8830 Da; expected = 8832.12 Da. UbBpa31, observed mass = 8961 Da; expected = 8963.32 Da. **h)** UbBpa32 (-Met), observed mass = 8845 Da; expected = 8845.17 Da. UbBpa32, observed mass = 8976 Da; expected = 8976.37 Da. **i)** UbBpa34 (-Met), observed mass = 8830 Da; expected = 8831.14 Da. UbBpa34, observed mass = 8961 Da; expected = 8962.34 Da. **j)** UbBpa40 (-Met), observed mass = 8831 Da; expected = 8832.12 Da. UbBpa40, observed mass = 8962 Da; expected = 8963.32 Da. **k)** UbBpa64 (-Met), observed mass = 8830 Da; expected = 8831.14 Da. UbBpa64, observed mass = 8961 Da; expected = 8962.34 Da. **l)** UbBpa72 (-Met), observed mass = 8803 Da; expected = 8804.07 Da. Observed peak at 8836 Da corresponds to an unidentified adduct.

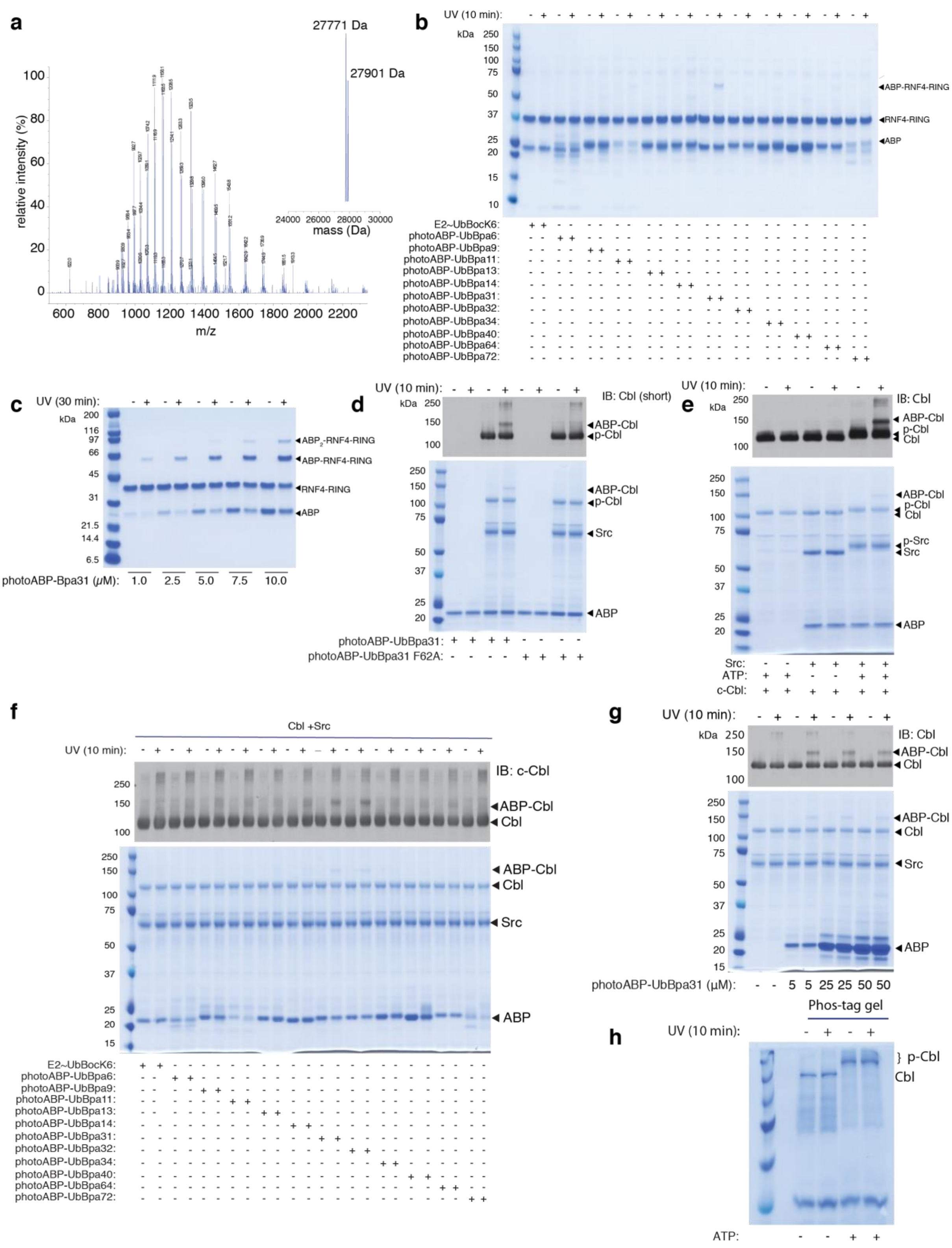

Figure S3. Supplemental photoABP characterization data, related to Figure 3 and 4. a) Raw ESI-MS spectrum for sample for photoABP-Bpa31. photoABP Bpa31 (-Met), observed mass = 27769 Da; expected mass = 27777.67 Da. photoABP-UbBpa31 (-Met), observed mass = 27901 Da; expected mass = 27908.87 Da. **b)** Assessment of RNF4-RING photocrosslinking efficiency for incorporation of Bpa at 10 different Ub positions within isopeptide-linked E2~Ub conjugate. Significant incorporation was only achieved with incorporation of Bpa at position 31, furnishing

probe photoABP-UbBpa31. The reference amino acid *t*-butyloxycarbonyl-L-lysine (BocK) and Bpa were incorporated at position 6 as this site is highly permissive to unnatural amino acid incorporation and served as positive controls (Virdee et al., 2010). **c)** Dose responsive photocrosslinking of RNF4-RING. Photocrosslinking efficiency of RNF4-RING was responsive to increasing concentrations of photoABP-UbBpa31. **d)** Photocrosslinking of phosphorylated c-Cbl with photoABP-UbBpa31 and the photoABP-UbBpa31 F62A control probe. Purified c-Cbl (3 μM) was preincubated with c-Src (1.5 μM) in the presence of ATP (5 mM) for 45 min at 37 ºC. Reaction mixture was then profiled with the specified probes (5 μM). The probe photoABP-Bpa31 undergoes c-Cbl crosslinking whereas photoABP-Bpa31 F62A does not. **e)** Photocrosslinking of Src treated c-Cbl is strictly ATP-dependent. Purified c-Cbl (3 μM) was preincubated with c-Src (1.5 μM) in the presence (5 mM) or absence of ATP for 45 min at 37 °C. Reaction mixture was then profiled with the specified probes (5 μM). The probe photoABP-Bpa31 undergoes c-Cbl crosslinking only in the presence of Src and ATP. **f)** Comparison of photocrosslinking efficiency for Bpa incorporation at multiple positions within ubiquitin. c-Cbl (3 μM) was phosphorylated by incubation with c-Src (1.5 μM) prior to probe analysis. Only incorporation of Bpa at positions 31 and 32 furnishes a functional probe. **g)** Dose-response analysis of Cbl crosslinking. c-Cbl (3 μM) was phosphorylated by incubation with c-Src (1.5 μM) prior to probe analysis. No increase in Cbl labelling efficiency was observed as a result of increasing photoABP-UbBpa31 concentration beyond 5 μM. **h)** Phostag SDS-PAGE analysis of c-Src treated c-Cbl. Reduced electrophoretic mobility of c-Cbl is only observed in the presence of ATP. As the gel shift is quantitative, this indicates that phosphorylation must too be quantitative. However, crosslinking efficiency with photoABP-UbBpa31 was not dose responsive beyond 5 μM. This is consistent with c-Cbl becoming phosphorylated at multiple sites but substoichiometrically at Y371.

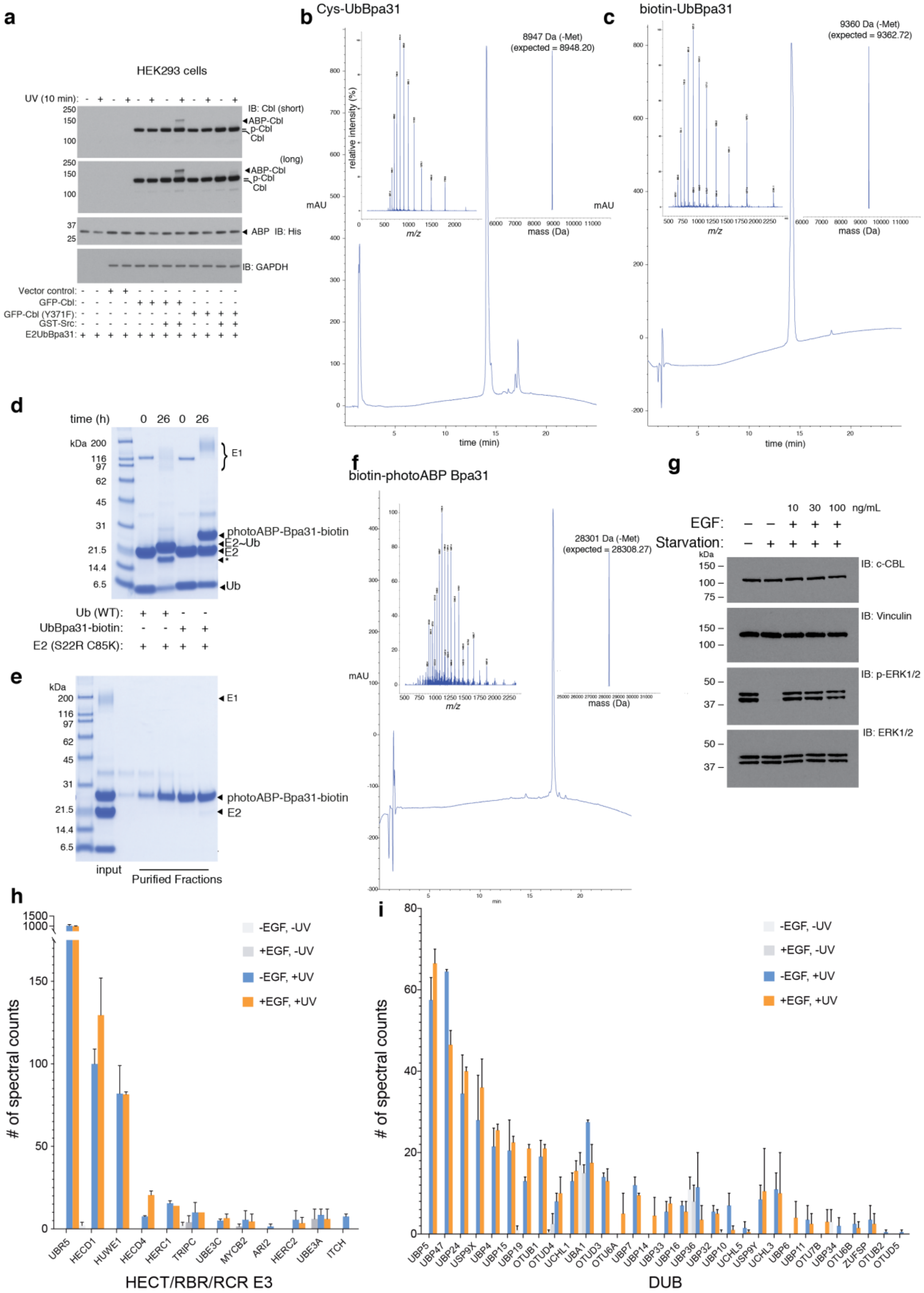

Figure S4. Supplemental blot for Figure 4b and characterization data for construction of biotinylated photoABP-Bpa31, confirmation of receptor tyrosine kinase activation and assessment of non-RING E3 photocrosslinking related to Figure 4. a)

Photocrosslinking of cellular Cbl remains strictly dependent on the presence of Y371. C-Cbl (3 μM) was phosphorylated by incubation with c-Src (1.5 μM) prior to probe analysis. **b)** Biotin labelling of Cys-tagged UbBpa31 and enzymatic conjugation to E2. LC-MS characterization of UbBpa31 expressed with an N-terminal MGCSSG labelling motif (observed mass = 8947 Da; expected mass = 8948.2 Da). **c)** Cysteine labelling motif was alkylated with EZ-Link Iodoacetyl-PEG2-Biotin (Thermofisher). Product was purified by preparative RP-HPLC and characterized by LC-MS (observed mass = 9360 Da; expected mass = 9362.72 Da). **d)** Refolded biotin-tagged UbBpa31 was enzymatically conjugated onto E2. **e)** The probe was purified by size-exclusion chromatography. **f)** LC-MS analysis for biotin-photoABP-Bpa31. HPLC chromatogram measured at 214 nm. photoABPBpa31 (-Met), observed mass = 28301 Da; expected mass = 28308.27 Da. **g)** EGF-dependent receptor activation is confirmed by immunoblotting for ERK1/2 phosphorylation. HEK293T cells were serum-starved and stimulated with recombinant EGF. Cells were treated with the proteasome inhibitor MG132 prior to stimulation. N.B. for proteomic experiment cells were treated with MG132 and bafilomycin. **h**) Detection of other ubiquitin system components by activity-based proteomics with biotinylated photoABP-UbBpa31. Spectral counts obtained from ABP-profiled HEK293T cells. Search results were filtered against the PFAM domain term "HECT, IBR and zf-UBR" and only RING E3s with >2 spectral counts in any replicate experiment were plotted. Cells were serum-starved and either treated with or without EGF and with or without UV irradiation. Errors bar correspond to the standard error from two technical replicates. **i**) As above but DUBs were filtered using a combination of PFAM domain terms and manual curation.

REAGENT or RESOURCE	SOURCE	IDENTIFIER
Oligonucleotides		
F Mut TAG9 (GATCTTCGTGAAGACCCTGtagGGTAAGACCATCACTCTCG) R mut TAG9 (CGAGAGTGATGGTCTTACCctaCAGGGTCTTCACGAAGATC)	This Manuscript	N/A
F Mut TAG11 (CGTGAAGACCCTGACTGGTtagACCATCACTCTCGAAGT) R mut TAG11 (ACTTCGAGAGTGATGGTctaACCAGTCAGGGTCTTCACG)	This Manuscript	N/A
F Mut TAG13 (GACCCTGACTGGTAAGACCtagACTCTCGAAGTGGAGCCGA) R mut TAG13 (TCGGCTCCACTTCGAGAGTctaGGTCTTACCAGTCAGGGTC)	This Manuscript	N/A
F Mut TAG14 (GACCCTGACTGGTAAGACCATCtagCTCGAAGTGGAGCCGAGTGACA) R mut TAG14 (TGTCACTCGGCTCCACTTCGAGctaGATGGTCTTACCAGTCAGGGTC)	This Manuscript	N/A
F Mut TAG31 (GAGAATGTCAAGGCAAAGATCtagGACAAGGAAGGCATCCCTCCT) R mut TAG31 (AGGAGGGATGCCTTCCTTGTCctaGATCTTTGCCTTGACATTCTC)	This Manuscript	N/A
F Mut TAG32 (GTCAAGGCAAAGATCCAAtagAAGGAAGGCATCCCTCCT) R mut TAG32 (AGGAGGGATGCCTTCCTTctaTTGGATCTTTGCCTTGAC)	This Manuscript	N/A
F Mut TAG34 (GCAAAGATCCAAGACAAGtagGGCATCCCTCCTGACCAG) R mut TAG34 (CTGGTCAGGAGGGATGCCctaCTTGTCTTGGATCTTTGC)	This Manuscript	N/A
F Mut TAG40 (GAAGGCATCCCTCCTGACtAGCAGAGGTTGATCTTTG) R mut TAG40 (CAAAGATCAACCTCTGCTaGTCAGGAGGGATGCCTTC)	This Manuscript	N/A
F Mut TAG64 (GACTACAACATCCAGAAAtAGTCCACCCTGCACCTGG) R mut TAG64 (CCAGGTGCAGGGTGGACTaTTTCTGGATGTTGTAGTC)	This Manuscript	N/A

F Mut TAG72<br>(CACCCTGCACCTGGTCCTCtagCTCCGAGGTGGGCATCACC)<br>R mut TAG72<br>(GGTGATGCCCACCTCGGAGctaGAGGACCAGGTGCAGGGTG)	This Manuscript	N/A
UbCH5C -S22R<br>NW2690<br>(GACCCTCCAGCACAATGTcgTGCAGGTCCAGTTGGGGA)<br>NW2691<br>(TCCCCAACTGGACCTGCAcgACATTGTGCTGGAGGGTC)<br>C85K<br>NW4685<br>(TAACAGTAATGGCAGCATTaaaCTCGATATTCTAAGATCAC)<br>NW4686<br>(GTGATCTTAGAATATCGAGtttAATGCTGCCATTACTGTTA)	This Manuscript	N/A
UbCH5C -F62A<br>NW4687<br>(CATTTTCCTACAGACTACCCCgcCAAACCACCTAAGGTTGCATT)<br>NW4688<br>(AATGCAACCTTAGGTGGTTTGgcGGGGTAGTCTGTAGGAAAATG)	This Manuscript	N/A
Cbl<br>NW6441<br>(CAGGAACAATATGAATTATtCTGTGAGATGGGCTCCACA)<br>NW6442<br>(TGTGGAGCCCATCTCACAGaATAATTCATATTGTTCCTG)	This Manuscript	N/A

Table S1. List of oligonucleotides generated in this study. Related to STAR Methods.
