# Supplementary material for: Photocrosslinking Activity-Based Probes for Ubiquitin RING E3 Ligases
Source: Cell Chem Biol. 2020 Jan 16;27(1):74–82.e6. doi: 10.1016/j.chembiol.2019.11.013 (PMC6963778; doi:10.1016/j.chembiol.2019.11.013)
Supplement: Document S2. Article plus Supplemental Information [file mmc2.pdf]

Brief Communication

Cell Chemical Biology

Photocrosslinking Activity-Based Probes for Ubiquitin RING E3 Ligases

Highlights

- Photoactivated activity-based probes developed for large class of ubiquitin E3 ligases
- ABPs are compatible with divergent RING E3 activation mechanisms
- Parallelized E3 profiling and detection of growth factor-induced E3 activation

Authors

Sunil Mathur, Adam J. Fletcher, Emma Branigan, Ronald T. Hay, Satpal Virdee

Correspondence

s.s.virdee@dundee.ac.uk

In Brief

Activity-based probes (ABPs) are valuable research tools for studying enzyme function. Ubiquitin E3 ligases are one of the largest enzyme families yet ABPs for this enzyme class do not exist. Mathur et al. developed photocrosslinking ABPs for RING E3s and using activity-based proteomics demonstrate activity-dependent readout of diverse E3 activation.

Mathur et al., 2020, Cell Chemical Biology 27, 74–82
January 16, 2020 © 2019 The Authors. Published by Elsevier Ltd.
https://doi.org/10.1016/j.chembiol.2019.11.013

CellPress
Cell Chemical Biology
Brief Communication

Photocrosslinking Activity-Based Probes for Ubiquitin RING E3 Ligases

Sunil Mathur,[1] Adam J. Fletcher,[1] Emma Branigan,[2] Ronald T. Hay,[2] and Satpal Virdee[1,3,*]
[1]MRC Protein Phosphorylation and Ubiquitylation Unit, University of Dundee, Scotland, UK
[2]Division of Gene Regulation and Expression, University of Dundee, Scotland, UK
[3]Lead Contact
*Correspondence: s.s.virdee@dundee.ac.uk
https://doi.org/10.1016/j.chembiol.2019.11.013

SUMMARY

Activity-based protein profiling is an invaluable technique for studying enzyme biology and facilitating the development of therapeutics. Ubiquitin E3 ligases (E3s) are one of the largest enzyme families and regulate a host of (patho)physiological processes. The largest subtype are the RING E3s of which there are >600 members. RING E3s have adaptor-like activity that can be subject to diverse regulatory mechanisms and have become attractive drug targets. Activity-based probes (ABPs) for measuring RING E3 activity do not exist. Here we re-engineer ubiquitin-charged E2 conjugating enzymes to produce photocrosslinking ABPs. We demonstrate activity-dependent profiling of two divergent cancer-associated RING E3s, RNF4 and c-Cbl, in response to their native activation signals. We also demonstrate profiling of endogenous RING E3 ligase activation in response to epidermal growth factor (EGF) stimulation. These photocrosslinking ABPs should advance E3 ligase research and the development of selective modulators against this important class of enzymes.

INTRODUCTION

Ubiquitination is fundamental posttranslational modification that regulates normal cellular physiology and its dysfunction can lead to disease onset (Rape, 2018). Ubiquitination is carried out by an enzymatic cascade involving the sequential activities of ubiquitin E1-activating (E1), ubiquitin E2-conjugating (E2), and ubiquitin E3 ligases (E3s) (Hershko and Ciechanover, 1998). Ubiquitin (Ub) is covalently transferred from catalytic cysteine in E1 onto catalytic cysteine in E2 forming a thioester-linked E2 intermediate (E2∼Ub). Hundreds of E3s are known to exist which recruit E2∼Ub and ubiquitinate-specific substrates. Divergence of E3 mechanism has led to two general classes. "Cys E3s," of which there are ∼50, utilize a catalytic cysteine to form a covalent thioester-linked intermediate with the Ub before substrate modification (Scheffner et al., 1995; Wenzel et al., 2011; Pao et al., 2018). However, the largest class are adapter-like E3s of which there are >600 distinct forms (Deshaies and Joazeiro, 2009). Adapter-like E3s are devoid of a catalytic nucleophile and catalyze direct transfer of Ub from E2∼Ub to substrate. This adapter-like activity is utilized by multi-subunit Cullin-RING E3s and ∼350 single polypeptide RING E3s (hereafter simply referred to as RING E3s). The latter can exist as monomers, homodimers, or heterodimers (Metzger et al., 2014). Activity regulation is a particularly important aspect of E3 biology that ensures cellular homeostasis and adaptive signaling. Dysregulation can lead to disease onset hence RING E3s have become attractive therapeutic targets (Burgess et al., 2016). However, the cellular roles and the regulatory mechanisms for the vast majority of RING E3s remain poorly understood. Furthermore, RING E3s have recently been shown to be compatible with targeted protein degradation strategies (e.g., PROTAC methodology) (Naito et al., 2019; Spradlin et al., 2019; Ward et al., 2019), so tools for determining which are active in clinical contexts are needed to further leverage this potential.

A hallmark of adapter-like E3s is that when in the active state, they shift the dynamic E2∼Ub conformational ensemble toward a distinct population where the E2∼Ub conjugate adopts a folded back or "closed" conformation (Pruneda et al., 2011, 2012; Dou et al., 2012b; Plechanovová et al., 2012) (Figure 1A). This conformation activates the thioester bond within E2∼Ub to nucleophilic attack and is a requisite for efficient aminolysis activity. RING E3 activity can be regulated and switching to an activated state is achieved by the E3 acquiring structural features that engage the Ub component thereby promoting induction of the closed conformation. For example, RING E3s such as RNF4 and BIRC7 are activated by RING domain dimerization where a tail region of the second RING protomer engages the Ub component (Dou et al., 2012b; Plechanovová et al., 2012). Dimerization can be regulated by cellular signals and in the case of RNF4, this is brought about by binding to poly-SUMO chains (Rojas-Fernandez et al., 2014).

For activation of monomeric RING E3s a so-called non-RING element has been shown to play a role in binding the Ub component and, in the case of Cbl-b and c-Cbl, this is a phosphorylated tyrosine residue (Dou et al., 2013). Phosphorylation is carried out by the kinase c-Src in response to growth factor stimulation and Cbl activation triggers the ubiquitination and degradation of receptor and non-receptor tyrosine kinases (Levkowitz et al., 1998, 1999; Yokouchi et al., 2001). RING E3s that require dimerization and the presence of a non-RING element have also been reported (Koliopoulos et al.,

 © 2019 The Authors. Published by Elsevier Ltd.
This is an open access article under the CC BY license (http://creativecommons.org/licenses/by/4.0/).

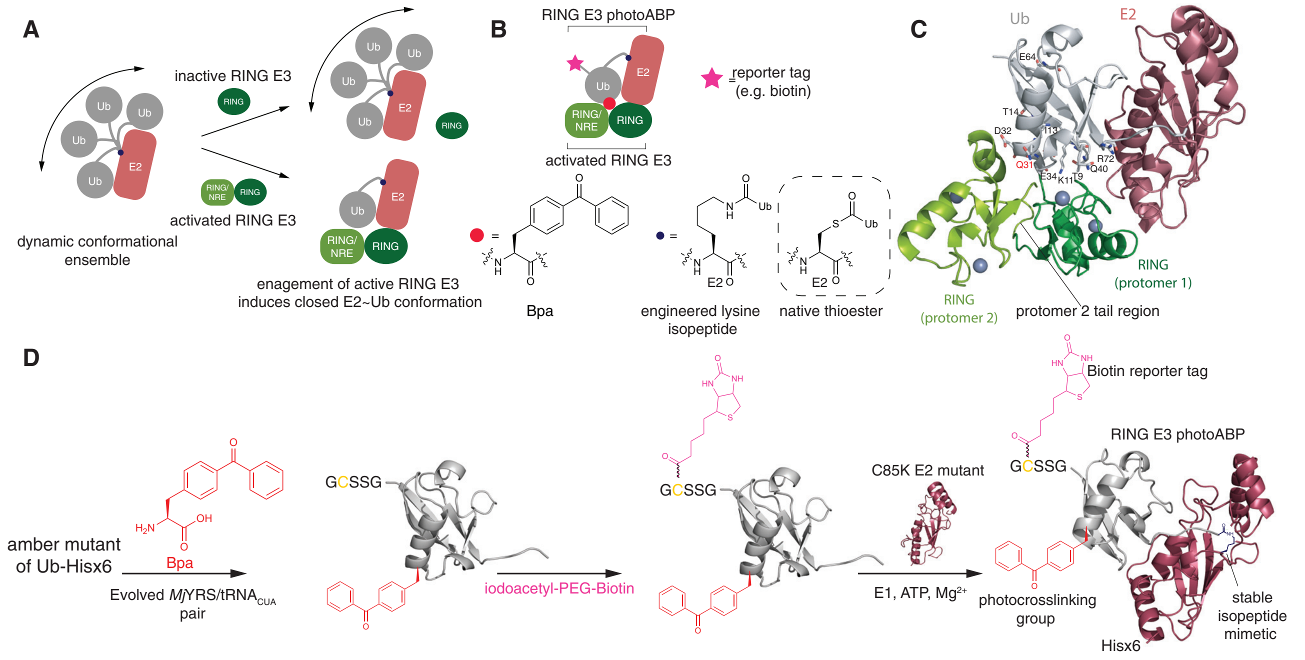

Figure 1. Strategy and Synthetic Scheme for Production of Photocrosslinking ABPs for RING E3 Ligases

(A) Binding of activated RING E3 induces closed conformation of otherwise conformationally dynamic E2∼Ub conjugate. Activation can be achieved by RING dimerization whereas monomeric RING E3s can be activated by the presence of a non-RING element (NRE). The Ub component of E2∼Ub interacts with RING/NRE region.

(B) Judicious incorporation of a *p*-benzoyl-L-phenyl alanine (Bpa) crosslinking amino acid within a stabilized E2∼Ub conjugate serves as an ABP for RING E3 activity.

(C) Crystal structure of E2∼Ub in complex with activated, dimeric RING E3 (RNF4; PDB: 4AP4). Ten amino acid sites within Ub that are proximal to the activated E3 were tested for Bpa incorporation. The Q31 (highlighted in red) was found to be optimal.

(D) Synthetic scheme for photocrosslinking ABP.

2016). Additional RING E3 activation mechanisms exist including allosteric binding of accessory proteins or ligands (DaRosa et al., 2015; Dickson et al., 2018; Duda et al., 2012; Wright et al., 2016). Numerous crystal structures of E2∼Ub bound to activated RING E3s have been solved revealing a highly conserved binding mode (Dou et al., 2012b, 2013; Koliopoulos et al., 2016; Plechanovová et al., 2012; Wright et al., 2016). Importantly, a consensus region of the Ub component in the closed E2∼Ub conjugate becomes proximal to the activated RING. Furthermore, biophysical analysis demonstrates that activated RING E3s studied thus far can have higher free energy of binding for E2∼Ub than their inactive forms (Berndsen et al., 2013; Buetow et al., 2016).

Activity-based probes (ABPs) are powerful chemical tools that undergo activity-dependent covalent labeling of enzyme family members (Niphakis and Cravatt, 2014; Hewings et al., 2017). This enables: (1) the study of enzyme regulation, (2) discovery of novel enzyme classes, (3) inhibitor screening, (4) inhibitor selectivity profiling, and (5) stabilization of enzymatic intermediates for structural studies (Hu et al., 2002). We and others have developed ABPs for Cys E3s which have been deployed to dissect E3 activation mechanisms and discover entirely novel E3 classes (Love et al., 2009; Pao et al., 2016, 2018; Mulder et al., 2016; Xu et al., 2019). ABPs that accurately assess RING E3 ligase activity do not currently exist.

We reasoned that the conserved (and activity-dependent) consensus interaction of the Ub component within the closed E2∼Ub, coupled with the enhanced free energy of binding for activated RING E3s, could be exploited for the development of ABPs for RING E3s. An ABP could then be based on an engineered E2∼Ub conjugate where the labile cysteine thioester has been replaced with a more stable linkage chemistry such as a lysine isopeptide (Plechanovová et al., 2012). However, ABPs typically contain an electrophilic warhead that covalently labels a catalytic nucleophile within the enzyme family under investigation. As RING E3s utilize an adapter-like mechanism, devoid of a catalytic nucleophile, we reasoned that rational placement of a photocrosslinking moiety within the E2∼Ub conjugate would compensate for the absence of this catalytic feature (Figure 1B). Such a strategy has been successfully employed for metalloenzymes that also lack a catalytic residue (Saghatelian et al., 2004). Incorporation of photocrosslinking groups into polymeric ubiquitin species have also been reported, where these serve as photoaffinity probes for generic Ub interactors (Chojnacki et al., 2017; Liang et al., 2017). The utility of photocrosslinker incorporation into E2s for E3 study has also been demonstrated. Crosslinker incorporation by chemical labeling has enabled the mapping of Cys E3 catalytic residues (Krist and Statsyuk, 2015) and, more recently, incorporation into an E2 by chemical synthesis has been used to generate a

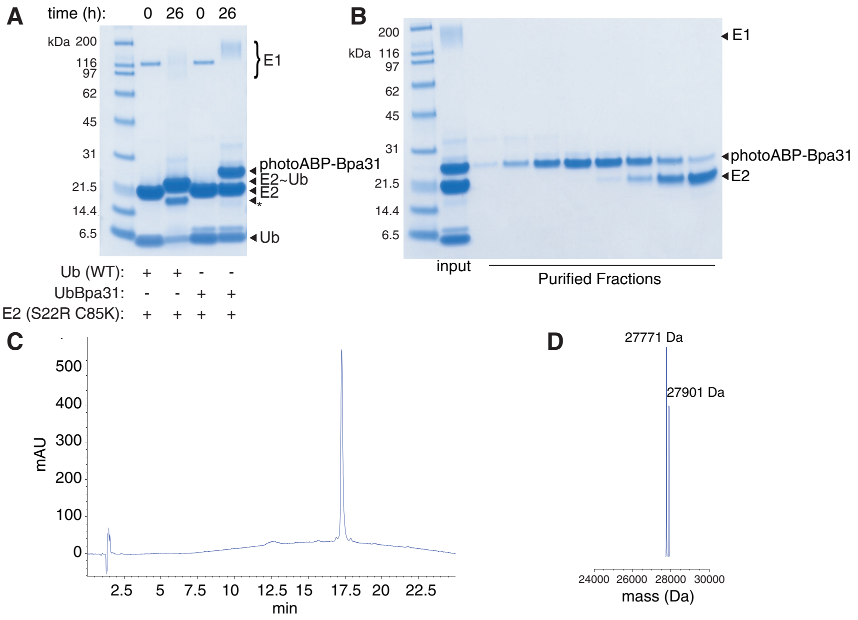

Figure 2. Assembly and Characterization of photoABP

(A) SDS-PAGE analysis of representative enzymatic conjugation of UbBpa31 to E2 (UBE2D3 C85K S22K double mutant) with ubiquitin E1 activating enzyme. Asterisk corresponds to a presumed diubiquitin species.

(B) Representative purification fractions of probe product after size-exclusion chromatography.

(C) Reverse-phase high-performance liquid chromatography chromatogram for purified photoABP-Bpa31.

(D) Deconvoluted mass spectrum for photoABP-Bpa31. Observed mass = 27,901 Da, observed mass (-Met) = 27,771 Da. Expected mass = 27,908.87 Da, expected mass (-Met) = 2,777.67 Da.

photoaffinity probe for a SUMO E3 (Zhang et al., 2019). However, the utility of these technologies as ABPs has not been demonstrated.

RESULTS

Design and Assembly of Photocrosslinking RING ABPs

To establish potential positions for photocrosslinker incorporation we generated structural superpositions for solved RING E3:E2∼Ub cocrystal structures (Figure S1) (Dou et al., 2012b, 2013; Koliopoulos et al., 2016; Plechanovová et al., 2012). Striking conservation in binding mode was apparent and multiple residues within both Ub and E2 lie proximal to the RING domain(s). To impart activity dependence to the probe we incorporated the photocrosslinking moiety into Ub as unlike the E2, this component only binds proximally to active RING E3s. We chose ten consensus sites within Ub that are proximal to activated RINGs (Figure 1C) and incorporated the photocrosslinking amino acid *p*-benzoyl-L-phenylalanine (Bpa) using an evolved *Methanocaldococcus jannaschii* tyrosyl-tRNA synthetase (*Mj*YRS)-tRNA$_{CUA}$ pair (Figures 1B and 1C) (Chin et al., 2002). Efficient incorporation was achieved at all sites yielding ∼4–6 mg of protein per liter of culture medium. Ub mutants were purified to homogeneity and characterized by liquid chromatography-mass spectrometry (LC-MS) (Figure S2). All of the mutant Ub variants were then enzymatically conjugated to the promiscuous E2 UBE2D3 (Brzovic and Klevit, 2006), bearing an N-terminal hexahistidine tag (Figure 1D). In addition to facilitating purification, the latter serves as a convenient reporter tag for immunoblot analysis. Conjugation to E2 was carried out with E1 activating enzyme and, to form a more stable linkage between Ub and E2, the catalytic cysteine was mutated to lysine enabling stable isopeptide conjugation (Plechanovová et al., 2012) (Figure 2A). Importantly, structural analysis has shown that the isopeptide is an acceptable structural mimetic of the native thioester (Koliopoulos et al., 2016; Plechanovová et al., 2012; Wright et al., 2016). We also introduced an S22R mutation into the E2 component, which disrupts a non-covalent Ub binding site that could result in ABP self-association (Brzovic et al., 2006). All E2∼Ub variants were purified to homogeneity by size-exclusion chromatography as determined by SDS-PAGE and LC-MS analysis (Figures 2B–2D and S3A).

Activity-Dependent Profiling of the Dimeric RING E3 RNF4

The RING E3 RNF4 is inactive in the monomeric state, which is predominant at endogenous concentrations. Binding of poly-SUMO chains to SUMO-interacting motifs (SIMs) within RNF4 enhances the local concentration of RNF4 thereby promoting RING domain homodimerization and activation of E3 ligase activity (Rojas-Fernandez et al., 2014). This leads to ubiquitination and degradation of SUMO-modified promyelocytic leukemia protein (Tatham et al., 2008). Strikingly, therapeutic induction of this process leads to remission of acute promyelocytic leukemia in >90% of cases (Massaro et al., 2016). An engineered version of RNF4 that is constitutively active has been designed that consists of full-length protein with an additional RING domain fused to the native C terminus via a flexible linker (RNF4-RING) (Figure 3A) (Plechanovová et al., 2011). To determine the optimum photocrosslinker position we incubated all ten Bpa mutant E2∼Ub conjugates with RNF4-RING (Figure S3B) and assessed crosslinking efficiency upon UV irradiation (10 min). Significant and dose-responsive crosslinking was only observed with Bpa incorporation at position 31 (photoABP-Bpa31) (Figures 3B and S3C). Importantly, no crosslinking was observed when RNF4-RING was incubated and UV irradiated with unconjugated UbBpa31, indicating that the photocrossslinking was dependent on E2-driven Ub proximity, consistent with the native mechanism. Notably, an additional crosslinking product corresponding to the molecular weight for the addition of two photoABP-Bpa31 molecules was observed (Figure 3B). Structural studies on dimeric RING:E2∼Ub complexes has revealed that both faces of the active RING dimer engage and activate a separate E2∼Ub conjugate. By virtue of the fused RNF4-RING construct it is possible to disrupt binding to a single E2∼Ub molecule, or both, by introducing an M140A

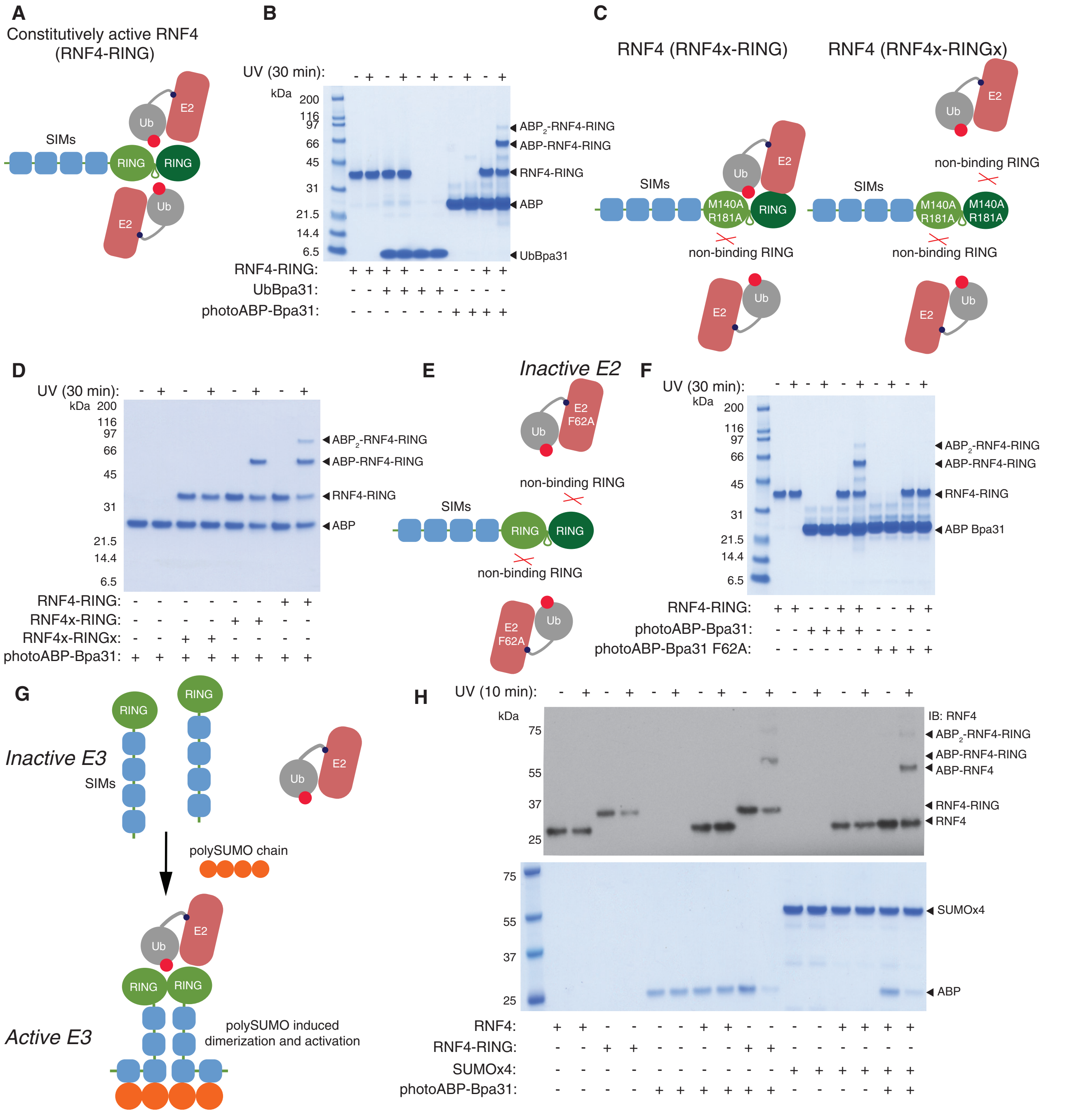

Figure 3. Activity-Dependent Profiling of RNF4 E3 Ligase Activity

(A) Constitutively active RNF-RING fusion protein can productively engage two E2~Ub conjugates.

(B) Probe photoABP-Bpa31 (40 μM) undergoes two crosslinking reactions with RNF4-RING (10 μM).

(C) Engagement of one or both E2~Ub conjugates can be disrupted with a M140A R181A double mutation introduced into one or both RING domains in RNF4-RING.

(D) Probe photoABP-Bpa31 (20 μM) crosslinking is attenuated or abolished depending on whether one or both RING domains are mutated.

(E) Introduction of a F62A mutation into the E2 component should abolish E3 binding.

(F) Crosslinking is abolished with the photoABP-Bpa31 F62A (probe concentration 40 μM).

(G) At concentrations below the K_d for dimerization, RNF4 is inactive. Binding of poly-SUMO chains induces dimerization and E3 ligase activity.

(H) photoABP-Bpa31 (5 μM) undergoes poly-SUMO chain (10 μM) dependent crosslinking of native RNF4 (100 nM) whereas RINF4-RING (50 nM) crosslinks independent of poly-SUMO chains. IB denotes immunoblot and the primary antibody used for detection is adjacent (i.e., anti-RNF4).

R181A double mutation into one or both of the RING domains (RNF4x-RING or RNF4x-RINGx, respectively) (Figure 3C) (Rojas-Fernandez et al., 2014). Consistent with photoABP-Bpa31 profiling this structurally elucidated bipartite mechanism, crosslinking of the second photoABP-Bpa31 molecule was lost with RNF4x-RING and was completely abolished with RNF4x-RINGx (Figure 3D). To further confirm activity-dependent photocrosslinking we prepared a mutant photoABP-Bpa31 control probe. Part of the conserved E2-RING interaction involves the E2 F62 residue (F63 in some model E2s) and mutation to alanine typically impairs/abolishes E3 binding (Figure 3E) (Weissman, 2001). This control probe would further inform on whether observed labeling is consistent with a native E2-RING interaction, thus suitable for screening inhibitors that disrupt the native interaction. Consistent with the probe being engaged in a native manner, the photoABP-Bpa31 F62A probe did not undergo RING crosslinking (Figure 3F). This probe should also serve as a valuable control when profiling RING E3s agnostically.

ABP Profiling of Poly-SUMO Chain-Induced RNF4 Activation

Cellular RNF4 is activated by recruitment to poly-SUMO chains via its SIM domains thereby inducing dimerization. The K_d of dimerization is ~180 nM (Rojas-Fernandez et al., 2014) so by working at concentrations below this value we established a biochemical assay to assess whether photoABP-Bpa31 could profile poly-SUMO chain-induced activation of native RNF4 (Figure 3G). As expected, constitutively active RNF4-RING was insensitive to dilution and underwent photoABP-Bpa31 crosslinking but native RNF4 did not (Figure 3H). However, in the presence of a linear amide-linked tetra-SUMO (SUMOx4) fusion protein (10 μM), which recapitulates the activation properties of native isopeptide-linked poly-SUMO chains (Tatham et al., 2008), photoABP-Bpa31 crosslinking was observed with an efficiency comparable with that of RNF4-RING. Insightfully, a crosslinked band was observed for addition of a second photoABP-Bpa31 molecule (Figure 3H). This suggests that natively activated wild-type RNF4 retains its bipartite activity and its associated processivity is presumably utilized in cells. Taken together, the data so far demonstrate that photoABP-Bpa31 undergoes activity-dependent crosslinking of a natively activated RING E3, which is devoid of a catalytic nucleophile.

Activity-Dependent Profiling of Phosphorylation-Induced RING E3 Activation

We next tested photoABP-Bpa31 with a RING E3 that activates via a distinct mechanism. The Cbl proteins are multidomain and multifunctional RING E3 ligases consisting of three homologs: c-Cbl, Cbl-b, and Cbl-c (Lyle et al., 2019). The majority of Cbl function is associated with RING E3 activity and involves regulation of angiogenesis and aberrations in Cbl activity have been implicated with a number of cancers. Cbl is overexpressed in many breast cancer cells and tissues and is also found to be downregulated in human myeloid neoplasms, and non-small-cell lung cancers (Kales et al., 2010; Tan et al., 2010). Hence, modulation of Cbl E3 activity is an attractive therapeutic strategy and has attracted considerable interest from the pharmaceutical industry. The most common mutation manifesting in the clinic is at residue Y371. Y371 is subject to growth factor-induced phosphorylation by c-Src kinase and this leads to structural changes that present a non-RING element, enhance affinity for E2~Ub, and stimulate Cbl E3 activity (Buetow et al., 2016; Dou et al., 2013). Indeed, the affinity for Cbl phosphorylated at Y371 (c-Cbl pTyr371) enhances E2~Ub affinity ~30-fold (Buetow et al., 2016).

To assess whether photoABP-Bpa31 can profile Src-dependent activation of c-Cbl E3 activity we incubated recombinant Src with Cbl and Cbl Y371F, the latter expected to be refractory to phosphorylation-induced activation. PhotoABP-Bpa31 crosslinking was observed for c-Cbl in the presence of c-Src but not for c-Cbl Y371F, nor when c-Src was withheld (Figure 4A). Labeling was again abolished with the photoABP-Bpa31 F62A control probe (Figure S3D), and was also ATP dependent (Figure S3E). Therefore, consistent with previous studies, phosphorylation at Y371 specifically is required for activation of E3 activity (Dou et al., 2013). We also tested the panel of engineered E2~Ub conjugates with Bpa incorporation at different positions against c-Src activated c-Cbl and found partial overlap of productive sites with those for RNF4 (Figure S3F). An optimal Bpa position was 31 but, unlike RNF4, position 32 also crosslinked with similar efficiency. This is perhaps reflective of nuances between the monomeric and dimeric activation mechanism exhibited by these two RING E3s (Dou et al., 2013; Plechanovová et al., 2012). Interestingly, photocrosslinking efficiency remained substoichiometric regardless of photoABP-Bpa31 concentration suggestive of a subpopulation of the recombinant protein preparation being active (Figure S3G). Although Phos-tag gel analysis indicated that Cbl was quantitatively phosphorylated (Figure S3H), Src is known to phosphorylate multiple sites within Cbl and the degree of probe labeling may reflect substoichiometric phosphorylation at position Y371 (Dou et al., 2012a). We tested if incubation with elevated concentrations of Src could enhance photocrosslinking efficiency but found that as concentrations approached stoichiometry, photocrosslinking was inhibited; presumably due to Src competition with photoABP-Bpa31 for Cbl binding (data not shown).

Profiling c-Cbl Activation in a Human Cell Line

To establish whether we could profile c-Cbl activation in a human cell line we transiently transfected HEK293 cells with GST-tagged c-Src (GST-Src) together with GFP-tagged c-Cbl (GFP-Cbl) or GFP-tagged c-Cbl Y371F (GFP-Cbl Y371F). To prevent potential degradation of activated Cbl due to autoubiquitination we treated cells with the proteasome inhibitor MG132 for 90 min before lysis. Activity-dependent crosslinking was strictly dependent on Src coexpression and the presence of Y371 but absent with the photoABP-Bpa31 F62A control probe (Figures 4B and S4A).

Profiling Endogenous RING E3 Activation in Response to Growth Factor Stimulation

We next assessed the ability to carry out parallelized profiling of endogenous RING E3 activation in response to a physiological stimulus. Such experiments would potentially enable poorly understood RING E3s to be ascribed to regulatory functions across a spectrum of both physiological and pathophysiological

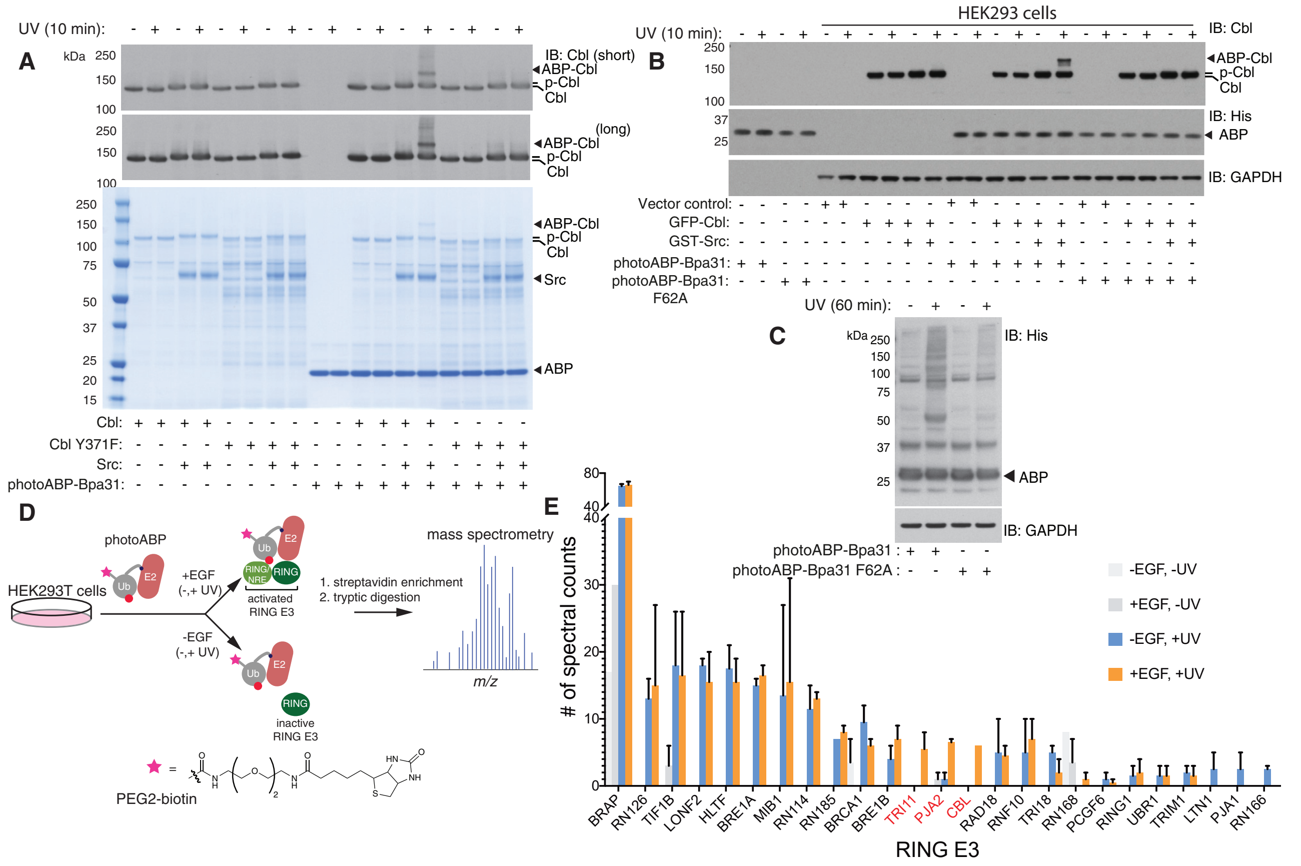

Figure 4. Activity-Dependent Profiling c-Cbl E3 Ligase Activity and Activity-Based Proteomic Analyses of EGF-Stimulated Versus Unstimulated HEK293T Cells

(A) Only c-Cbl (3 μM) preincubated with c-Src (1.5 μM) undergoes photoABP-Bpa31 (5 μM) crosslinking. Crosslinking is not observed when Cbl Y371 (3 μM), which cannot be phosphorylated at the activation site, is incubated with Src. Phosphorylation of Cbl results in reduced electrophoretic mobility.

(B) Transient overexpression of GFP-Cbl and c-Src in mammalian HEK293 cells. Extracts were treated with photoABP-Bpa31 or the F62A control probe (5 μM). IB denotes immunoblot and the primary antibody used for detection is adjacent (i.e., anti-Cbl).

(C) Immunoblot analysis of HEK293T extracts with either photoABP-Bpa31 or the photoABP-Bpa31 F62A control probe (10 μM). Blotting was carried out against the hexahistidine reporter tag present within the ABPs. Samples were irradiated for 60 min or irradiation was withheld.

(D) Schematic depicting activity-based proteomic workflow with biotinylated photoABP-Bpa31.

(E) Spectral counts obtained from ABP-profiled HEK293T cells. Search results were filtered against the PFAM domain term "RING" and only RING E3s with >2 spectral counts in any replicate experiment were plotted. Cells were serum-starved and either treated with or without EGF and with or without UV irradiation. Errors bar correspond to the standard error from two technical replicate LC-MS/MS analyses.

processes. However, depletion of the ABP by promiscuous crosslinking might compromise RING E3 coverage and was initially tested for by immunoblotting against the hexahistidine reporter tag, which proved not to be the case (Figure 4C). Furthermore, crosslinking was substantially reduced with the photoABP-Bpa31 F62A control probe, thereby implying that many of the crosslinked proteins are likely to be E3s (Figure 4C).

We prepared a biotinylated variant of photoABP-Bpa31 allowing selective enrichment of crosslinked proteins from complex cellular samples. Bpa was incorporated into N-terminal cysteine-tagged Ub and labeled with idodoacetyl-PEG2-biotin (Pao et al., 2018). Biotin labeled UbBpa31 was then enzymatically conjugated to E2 via an isopeptide bond using the procedure for untagged Ub (Figures S4B–S4F). We next tested whether endogenous Cbl activation could be detected in response to epidermal growth factor (EGF) stimulation, which induces Cbl phosphorylation (Levkowitz et al., 1998, 1999). HEK293T cells were stimulated with EGF and, to prevent potential degradation of activated RING E3s, we prior treated with the proteasome and lysosomal inhibitors MG132 and bafilomycin, respectively. Parallel experiments confirmed EGF responsiveness by immunoblotting for downstream mitogen-activated protein kinase activation, which is a robust marker of EGF receptor activation (Traverse et al., 1992) (Figure S4G).

Extracted proteomes were incubated with biotinylated photoABP-Bpa31 and enriched against streptavidin resin (Pao et al., 2018) (Figure 4D). Identification of crosslinked proteins and their probe reactivity was inferred by streptavidin enrichment followed by data-dependent LC-tandem MS (LC-MS/MS) and spectral counting (Pao et al., 2018). Twenty-five RING E3s were detected, including Cbl, and Cbl peptides were only detected in EGF and UV treated samples (Figure 4E). This suggests that the

photocrosslinking probe can detect native RING E3 activation at the endogenous level. Interestingly, there was a notable increase in spectral counts for two other RING E3s, Praja2 and TRIM11 that was EGF and UV dependent (Figure 4E). As both of these E3s have been implicated with growth factor signaling, their detection may also be reflective of their activation or upregulation in response to EGF stimulation (Di et al., 2013; Rinaldi et al., 2016).

As Ub-interacting surfaces are shared across various ubiquitin system enzymes, we unexpectedly obtained UV-dependent enrichment of HECT (11), RBR (1), and RCR (1) E3s, as well as deubiquitinating enzymes (31) and an E1-activating enzyme (Figures S4H and S4I). As a consequence, probe modification of these additional ubiquitin system components could modulate their activity and in turn alter the activation status, or stability, of RING E3s under investigation. However, this is unlikely to pose any limitations beyond those associated with the employment of cellular extracts where the majority of cellular processes would be arrested.

DISCUSSION

In summary, we have developed activity-based probes for the adapter-like activity of RING E3 ligases. We demonstrate an activity-dependent signal for RNF4 and c-Cbl in response to their native activation cues and how the ABP-based readout can afford further mechanistic insights. These tools allow direct assessment of RING E3 activity (no dependence on E1, E2, or substrate) in diverse sample types. We also demonstrate parallelized profiling of a subset of endogenous RING E3s in extracted proteomes and detect activation of Cbl in response to growth factor stimulation. Therefore, this technology should find utility in the study of RING E3 regulatory biology, target discovery, biomarker applications, and modulator discovery. Detection of only a subset of RING E3s in our LC-MS/MS experiments might be reflective of many being inactive or beyond the detection limit of our current experimental conditions. Another possibility is that many E3s are not functional with the E2 enzyme photoABP-Bpa31 is based on (UBE2D3). However, the engineered isopeptide conjugation strategy, for stabilizing the labile thioester, has been demonstrated with E2s that are divergent from the E2 UBE2D3 employed herein (Branigan et al., 2015; Ordureau et al., 2015). Hence, our highly modular probe production strategy should be readily applicable to other E2s simply by using distinct recombinant E2 building blocks. This would potentially grant broader RING E3 coverage and also provide insights into cellular E2-E3 interaction networks.

SIGNIFICANCE

The ubiquitin system regulates a host of cellular process in both health and disease. Hundreds of single polypeptide RING E3s are encoded by the human genome, yet the cellular roles and how activity is regulated has only been demonstrated for a handful of members. Agnostic assessment of the activation status of RING E3s, using the reported photoABP technology, should facilitate the elucidation of RING E3 biological function and the discovery of novel regulatory mechanisms. RING E3s have also become highly attractive therapeutic targets. However, there is a paucity of methods for measuring RING E3 activity without convoluted reconstitution of the E1-E2-E3 cascade. Assessment of endogenous E3 activity can also be challenging. The photoABPs might allow the development of simplified and direct assays for the discovery of therapeutic RING E3 modulators. The ability to parallelize measurements at the endogenous level by interfacing with mass spectrometry could also facilitate comprehensive selective profiling of therapeutic candidates. RING E3s have also recently been shown to be amenable to targeted protein degradation strategies. To fully realize this potential it is important to establish which RING E3s might be active in certain cell types. Here, the photoABPs may find further utility.

STAR★METHODS

Detailed methods are provided in the online version of this paper and include the following:

- KEY RESOURCES TABLE
- LEAD CONTACT AND MATERIALS AVAILABILITY
- EXPERIMENTAL MODEL AND SUBJECT DETAILS
- METHODS DETAILS
  - Site-Specific Incorporation of *p*Bpa Unnatural Amino Acid into Ubiquitin
  - Expression of UBE2D3(S22R/C85K) Recombinant Protein
  - Preparation of Biotin-UbBpa31
  - Preparation of Isopeptide-linked photoABPs
  - Expression of Recombinant RNF4 Protein
  - Expression of c-Cbl and c-Cbl (Y371F) Recombinant Protein
  - c-Cbl Phosphorylation
  - UV Irradiation Conditions for Photo-Cross-Linking
  - Photo-Crosslinking in Cell Extracts
  - Phos-tag™ Gel Electrophoresis
  - Cell Culture, Transfection and Lysis
  - Activity-Based Proteomic Profiling of EGF-Stimulated HEK293 Cells
- QUANTIFICATION AND STATISTICAL ANALYSIS
- DATA AND CODE AVAILBILITY

SUPPLEMENTAL INFORMATION

Supplemental Information can be found online at https://doi.org/10.1016/j.chembiol.2019.11.013.

ACKNOWLEDGMENTS

We thank N. Wood of the MRC PPU Cloning Facility, J. Varghese of the MRC PPU Proteomics Facility, and the MRC PPU DNA Sequencing Facility. This work was funded by the Biotechnology and Biological Sciences Research Council (BB/P003982/1); UK Medical Research Council (MC_UU_12016/8); Wellcome Trust, United Kingdom, Senior Investigator award (098391/Z/12/Z) to R.T.H.; pharmaceutical companies supporting the Division of Signal Transduction Therapy (Boehringer-Ingelheim, GlaxoSmithKline, United Kingdom and Merck KGaA, Germany).

AUTHOR CONTRIBUTIONS

S.M., A.J.F., R.T.H., and S.V. designed the research. S.M. constructed probes and validated against purified proteins with assistance from E.B. S.M. and

A.J.F. carried out profiling experiments on cellular extracts. S.V. wrote the manuscript with input from all authors.

DECLARATION OF INTERESTS

S.V. and S. M. are authors of a patent related to the described probe technology.

Received: July 22, 2019
Revised: October 13, 2019
Accepted: November 20, 2019
Published: December 16, 2019

REFERENCES

Berndsen, C.E., Wiener, R., Yu, I.W., Ringel, A.E., and Wolberger, C. (2013). A conserved asparagine has a structural role in ubiquitin-conjugating enzymes. Nat. Chem. Biol. *9*, 154–156.

Branigan, E., Plechanovova, A., Jaffray, E.G., Naismith, J.H., and Hay, R.T. (2015). Structural basis for the RING-catalyzed synthesis of K63-linked ubiquitin chains. Nat. Struct. Mol. Biol. *22*, 597–602.

Brzovic, P.S., and Klevit, R.E. (2006). Ubiquitin transfer from the E2 perspective: why is UbcH5 so promiscuous? Cell Cycle *5*, 2867–2873.

Brzovic, P.S., Lissounov, A., Christensen, D.E., Hoyt, D.W., and Klevit, R.E. (2006). A UbcH5/ubiquitin noncovalent complex is required for processive BRCA1-directed ubiquitination. Mol. Cell *21*, 873–880.

Buetow, L., Tria, G., Ahmed, S.F., Hock, A., Dou, H., Sibbet, G.J., Svergun, D.I., and Huang, D.T. (2016). Casitas B-lineage lymphoma linker helix mutations found in myeloproliferative neoplasms affect conformation. BMC Biol. *14*, 76.

Burgess, A., Chia, K.M., Haupt, S., Thomas, D., Haupt, Y., and Lim, E. (2016). Clinical overview of MDM2/X-targeted therapies. Front. Oncol. *6*, 7.

Chin, J.W., Martin, A.B., King, D.S., Wang, L., and Schultz, P.G. (2002). Addition of a photocrosslinking amino acid to the genetic code of Escherichiacoli. Proc. Natl. Acad. Sci. U S A *99*, 11020–11024.

Chojnacki, M., Mansour, W., Hameed, D.S., Singh, R.K., El Oualid, F., Rosenzweig, R., Nakasone, M.A., Yu, Z., Glaser, F., Kay, L.E., et al. (2017). Polyubiquitin-photoactivatable crosslinking reagents for mapping ubiquitin interactome identify Rpn1 as a proteasome ubiquitin-associating subunit. Cell Chem. Biol. *24*, 443–457.e6.

DaRosa, P.A., Wang, Z., Jiang, X., Pruneda, J.N., Cong, F., Klevit, R.E., and Xu, W. (2015). Allosteric activation of the RNF146 ubiquitin ligase by a poly(ADP-ribosyl)ation signal. Nature *517*, 223–226.

Deshaies, R.J., and Joazeiro, C.A. (2009). RING domain E3 ubiquitin ligases. Annu. Rev. Biochem. *78*, 399–434.

Di, K., Linskey, M.E., and Bota, D.A. (2013). TRIM11 is overexpressed in high-grade gliomas and promotes proliferation, invasion, migration and glial tumor growth. Oncogene *32*, 5038–5047.

Dickson, C., Fletcher, A.J., Vaysburd, M., Yang, J.C., Mallery, D.L., Zeng, J., Johnson, C.M., McLaughlin, S.H., Skehel, M., Maslen, S., et al. (2018). Intracellular antibody signalling is regulated by phosphorylation of the Fc receptor TRIM21. Elife *7*, https://doi.org/10.7554/eLife.32660.

Dou, H., Buetow, L., Hock, A., Sibbet, G.J., Vousden, K.H., and Huang, D.T. (2012a). Structural basis for autoinhibition and phosphorylation-dependent activation of c-Cbl. Nat. Struct. Mol. Biol. *19*, 184–192.

Dou, H., Buetow, L., Sibbet, G.J., Cameron, K., and Huang, D.T. (2012b). BIRC7-E2 ubiquitin conjugate structure reveals the mechanism of ubiquitin transfer by a RING dimer. Nat. Struct. Mol. Biol. *19*, 876–883.

Dou, H., Buetow, L., Sibbet, G.J., Cameron, K., and Huang, D.T. (2013). Essentiality of a non-RING element in priming donor ubiquitin for catalysis by a monomeric E3. Nat. Struct. Mol. Biol. *20*, 982–986.

Duda, D.M., Olszewski, J.L., Tron, A.E., Hammel, M., Lambert, L.J., Waddell, M.B., Mittag, T., DeCaprio, J.A., and Schulman, B.A. (2012). Structure of a glomulin-RBX1-CUL1 complex: inhibition of a RING E3 ligase through masking of its E2-binding surface. Mol. Cell *47*, 371–382.

Hershko, A., and Ciechanover, A. (1998). The ubiquitin system. Annu. Rev. Biochem. *67*, 425–479.

Hewings, D.S., Flygare, J.A., Bogyo, M., and Wertz, I.E. (2017). Activity-based probes for the ubiquitin conjugation-deconjugation machinery: new chemistries, new tools, and new insights. FEBS J. *284*, 1555–1576.

Hu, M., Li, P., Li, M., Li, W., Yao, T., Wu, J.W., Gu, W., Cohen, R.E., and Shi, Y. (2002). Crystal structure of a UBP-family deubiquitinating enzyme in isolation and in complex with ubiquitin aldehyde. Cell *111*, 1041–1054.

Kales, S.C., Ryan, P.E., Nau, M.M., and Lipkowitz, S. (2010). Cbl and human myeloid neoplasms: the Cbl oncogene comes of age. Cancer Res. *70*, 4789–4794.

Koliopoulos, M.G., Esposito, D., Christodoulou, E., Taylor, I.A., and Rittinger, K. (2016). Functional role of TRIM E3 ligase oligomerization and regulation of catalytic activity. EMBO J. *35*, 1204–1218.

Krist, D.T., and Statsyuk, A.V. (2015). Catalytically important residues of E6AP ubiquitin ligase identified using acid-cleavable photo-cross-linkers. Biochemistry *54*, 4411–4414.

Levkowitz, G., Waterman, H., Ettenberg, S.A., Katz, M., Tsygankov, A.Y., Alroy, I., Lavi, S., Iwai, K., Reiss, Y., Ciechanover, A., et al. (1999). Ubiquitin ligase activity and tyrosine phosphorylation underlie suppression of growth factor signaling by c-Cbl/Sli-1. Mol. Cell *4*, 1029–1040.

Levkowitz, G., Waterman, H., Zamir, E., Kam, Z., Oved, S., Langdon, W.Y., Beguinot, L., Geiger, B., and Yarden, Y. (1998). c-Cbl/Sli-1 regulates endocytic sorting and ubiquitination of the epidermal growth factor receptor. Genes Dev. *12*, 3663–3674.

Liang, J., Zhang, L., Tan, X.L., Qi, Y.K., Feng, S., Deng, H., Yan, Y., Zheng, J.S., Liu, L., and Tian, C.L. (2017). Chemical synthesis of diubiquitin-based photo-affinity probes for selectively profiling ubiquitin-binding proteins. Angew. Chem. Int. Ed. *56*, 2744–2748.

Love, K.R., Pandya, R.K., Spooner, E., and Ploegh, H.L. (2009). Ubiquitin C-terminal electrophiles are activity-based probes for identification and mechanistic study of ubiquitin conjugating machinery. ACS Chem. Biol. *4*, 275–287.

Lyle, C.L., Belghasem, M., and Chitalia, V.C. (2019). c-Cbl: an important regulator and a target in angiogenesis and tumorigenesis. Cells *8*, https://doi.org/10.3390/cells8050498.

Massaro, F., Molica, M., and Breccia, M. (2016). Current first- and second-line treatment options in acute promyelocytic leukemia. Int. J. Hematol. Oncol. *5*, 105–118.

Metzger, M.B., Pruneda, J.N., Klevit, R.E., and Weissman, A.M. (2014). RING-type E3 ligases: master manipulators of E2 ubiquitin-conjugating enzymes and ubiquitination. Biochim. Biophys. Acta *1843*, 47–60.

Mulder, M.P., Witting, K., Berlin, I., Pruneda, J.N., Wu, K.P., Chang, J.G., Merkx, R., Bialas, J., Groettrup, M., Vertegaal, A.C., et al. (2016). A cascading activity-based probe sequentially targets E1-E2-E3 ubiquitin enzymes. Nat. Chem. Biol. *12*, 523–530.

Naito, M., Ohoka, N., and Shibata, N. (2019). SNIPERs-Hijacking IAP activity to induce protein degradation. Drug Discov. Today Technol. *31*, 35–42.

Niphakis, M.J., and Cravatt, B.F. (2014). Enzyme inhibitor discovery by activity-based protein profiling. Annu. Rev. Biochem. *83*, 341–377.

Ordureau, A., Heo, J.M., Duda, D.M., Paulo, J.A., Olszewski, J.L., Yanishevski, D., Rinehart, J., Schulman, B.A., and Harper, J.W. (2015). Defining roles of PARKIN and ubiquitin phosphorylation by PINK1 in mitochondrial quality control using a ubiquitin replacement strategy. Proc. Natl. Acad. Sci. U S A *112*, 6637–6642.

Pao, K.C., Stanley, M., Han, C., Lai, Y.C., Murphy, P., Balk, K., Wood, N.T., Corti, O., Corvol, J.C., Muqit, M.M., et al. (2016). Probes of ubiquitin E3 ligases enable systematic dissection of parkin activation. Nat. Chem. Biol. *12*, 324–331.

Pao, K.C., Wood, N.T., Knebel, A., Rafie, K., Stanley, M., Mabbitt, P.D., Sundaramoorthy, R., Hofmann, K., van Aalten, D.M.F., and Virdee, S. (2018). Activity-based E3 ligase profiling uncovers an E3 ligase with esterification activity. Nature *556*, 381–385.

CellPress

Plechanovová, A., Jaffray, E.G., McMahon, S.A., Johnson, K.A., Navratilova, I., Naismith, J.H., and Hay, R.T. (2011). Mechanism of ubiquitylation by dimeric RING ligase RNF4. Nat. Struct. Mol. Biol. *18*, 1052–1059.

Plechanovová, A., Jaffray, E.G., Tatham, M.H., Naismith, J.H., and Hay, R.T. (2012). Structure of a RING E3 ligase and ubiquitin-loaded E2 primed for catalysis. Nature *489*, 115–120.

Pruneda, J.N., Littlefield, P.J., Soss, S.E., Nordquist, K.A., Chazin, W.J., Brzovic, P.S., and Klevit, R.E. (2012). Structure of an E3:E2∼Ub complex reveals an allosteric mechanism shared among RING/U-box ligases. Mol. Cell *47*, 933–942.

Pruneda, J.N., Stoll, K.E., Bolton, L.J., Brzovic, P.S., and Klevit, R.E. (2011). Ubiquitin in motion: structural studies of the ubiquitin-conjugating enzyme approximately ubiquitin conjugate. Biochemistry *50*, 1624–1633.

Rape, M. (2018). Ubiquitylation at the crossroads of development and disease. Nat. Rev. Mol. Cell Biol. *19*, 59–70.

Rinaldi, L., Delle Donne, R., Sepe, M., Porpora, M., Garbi, C., Chiuso, F., Gallo, A., Parisi, S., Russo, L., Bachmann, V., et al. (2016). praja2 regulates KSR1 stability and mitogenic signaling. Cell Death Dis. *7*, e2230.

Rojas-Fernandez, A., Plechanovova, A., Hattersley, N., Jaffray, E., Tatham, M.H., and Hay, R.T. (2014). SUMO chain-induced dimerization activates RNF4. Mol. Cell *53*, 880–892.

Saghatelian, A., Jessani, N., Joseph, A., Humphrey, M., and Cravatt, B.F. (2004). Activity-based probes for the proteomic profiling of metalloproteases. Proc. Natl. Acad. Sci. U S A *101*, 10000–10005.

Scheffner, M., Nuber, U., and Huibregtse, J.M. (1995). Protein ubiquitination involving an E1-E2-E3 enzyme ubiquitin thioester cascade. Nature *373*, 81–83.

Spradlin, J.N., Hu, X., Ward, C.C., Brittain, S.M., Jones, M.D., Ou, L., To, M., Proudfoot, A., Ornelas, E., Woldegiorgis, M., et al. (2019). Harnessing the anti-cancer natural product nimbolide for targeted protein degradation. Nat. Chem. Biol. *15*, 747–755.

Tan, Y.H., Krishnaswamy, S., Nandi, S., Kanteti, R., Vora, S., Onel, K., Hasina, R., Lo, F.Y., El-Hashani, E., Cervantes, G., et al. (2010). CBL is frequently altered in lung cancers: its relationship to mutations in MET and EGFR tyrosine kinases. PLoS One *5*, e8972.

Tatham, M.H., Geoffroy, M.C., Shen, L., Plechanovova, A., Hattersley, N., Jaffray, E.G., Palvimo, J.J., and Hay, R.T. (2008). RNF4 is a poly-SUMO-specific E3 ubiquitin ligase required for arsenic-induced PML degradation. Nat. Cell Biol. *10*, 538–546.

Traverse, S., Gomez, N., Paterson, H., Marshall, C., and Cohen, P. (1992). Sustained activation of the mitogen-activated protein (MAP) kinase cascade may be required for differentiation of PC12 cells. Comparison of the effects of nerve growth factor and epidermal growth factor. Biochem. J. *288* (Pt 2), 351–355.

Virdee, S., Ye, Y., Nguyen, D.P., Komander, D., and Chin, J.W. (2010). Engineered diubiquitin synthesis reveals Lys29-isopeptide specificity of an OTU deubiquitinase. Nat. Chem. Biol. *6*, 750–757.

Ward, C.C., Kleinman, J.I., Brittain, S.M., Lee, P.S., Chung, C.Y.S., Kim, K., Petri, Y., Thomas, J.R., Tallarico, J.A., McKenna, J.M., et al. (2019). Covalent ligand screening uncovers a RNF4 E3 ligase recruiter for targeted protein degradation applications. ACS Chem. Biol. *14*, 2430–2440.

Weissman, A.M. (2001). Themes and variations on ubiquitylation. Nat. Rev. Mol. Cell Biol. *2*, 169–178.

Wenzel, D.M., Lissounov, A., Brzovic, P.S., and Klevit, R.E. (2011). UBCH7 reactivity profile reveals parkin and HHARI to be RING/HECT hybrids. Nature *474*, 105–108.

Wright, J.D., Mace, P.D., and Day, C.L. (2016). Secondary ubiquitin-RING docking enhances Arkadia and Ark2C E3 ligase activity. Nat. Struct. Mol. Biol. *23*, 45–52.

Xu, L., Fan, J., Wang, Y., Zhang, Z., Fu, Y., Li, Y.M., and Shi, J. (2019). An activity-based probe developed by a sequential dehydroalanine formation strategy targets HECT E3 ubiquitin ligases. Chem. Commun. (Camb.) *55*, 7109–7112.

Yokouchi, M., Kondo, T., Sanjay, A., Houghton, A., Yoshimura, A., Komiya, S., Zhang, H., and Baron, R. (2001). Src-catalyzed phosphorylation of c-Cbl leads to the interdependent ubiquitination of both proteins. J. Biol. Chem. *276*, 35185–35193.

Young, T.S., Ahmad, I., Yin, J.A., and Schultz, P.G. (2010). An enhanced system for unnatural amino acid mutagenesis in *E. coli*. J. Mol. Biol. *395*, 361–374.

Zhang, Y., Hirota, T., Kuwata, K., Oishi, S., Gramani, S.G., and Bode, J.W. (2019). Chemical synthesis of atomically tailored SUMO E2 conjugating enzymes for the formation of covalently linked SUMO-E2-E3 ligase ternary complexes. J. Am. Chem. Soc. *141*, 14742–14751.

STAR★METHODS

KEY RESOURCES TABLE

REAGENT or RESOURCE	SOURCE	IDENTIFIER
**Antibodies**		
Cbl mouse monoclonal antibody	Santa Cruz Biotechnology	Cat#Sc-1651; RRID: AB_2244054
6XHis mouse monoclonal antibody	Clonetech	Cat#631212; RRID: AB_2721905
Anti-mouse antibody IgG, HRP-linked	Cell-signalling Technology	Cat#7076S; RRID: AB_330924
RNF4 monoclonal antibody	Ronald T. Hay	University of Dundee
GAPDH mouse monoclonal antibody	Proteintech	Cat#60004-1-Ig; RRID: AB_2107436
p-ERK1/2	Cell-signalling Technology	Cat#9101; RRID: AB_331646
ERK1/2	Cell-signalling Technology	Cat#9102; RRID: AB_330744
**Bacterial and Virus Strains**		
BL21 DE3	Merck	Cat#69450
Rosetta (DE3) cells- Novagen	Merck	Cat#7054
**Chemicals, Peptides, and Recombinant Proteins**		
p-Benzoyl-L-phenylalanine (Bpa)	Bachem	Cat#4017646.0005
t-butyloxycarbonyl-L-lysine (BocK)	Bachem	Cat#4000211.0025
BugBuster® Protein Extraction	Merck Millipore	Cat#70584
MES-SDS Running Buffer 20X	FORMEDIUM™	Cat#MES-SDS5000
MOPS-SDS Running Buffer 20X	ThermoFisher Scientific	Cat#NP0001
InstantBlue™ Protein Stain	Expedeon	Cat#ISB1L
His-UBE1	MRC Reagents and Services University of Dundee	DU32888
UCH-L3 (N-terminal GST)	MRC Reagents and Services University of Dundee	DU21015
TEV protease	MRC Reagents and Services University of Dundee	DU6811
Src (1-536)	MRC Reagents and Services University of Dundee	DU19041
Recombinant epidermal growth factor	ThermoFisher Scientific	Cat#PHG0311
cOmplete protease inhibitor cocktail (EDTA-free)	Roche Diagnostics GmbH	Cat#11873580001
Lysozyme	Sigma	Cat#L6876
Nickel-NTA agarose	MRC Reagents and Services University of Dundee	https://mrcintranet.lifesci.dundee.ac.uk/mrc-reagents
Glutathione sepharose 4B	GE Life Sciences	#GE17-0756-01
EZ-link iodo-acetyl PEG2-Biotin	ThermoFisher Scientific	Cat#21334
NuPAGE™ LDS Sample Buffer (4X)	ThermoFisher Scientific	Cat#NP0007
β-mercaptoethanol	Sigma-Aldrich	Cat#M6250
Sodium Chloride	VMR Chemicals	Cat#1310-73-2
Dithiothreitol (DTT)	Melford	Cat#D11000
Trifluoroacetic acid (TFA)	Sigma-Aldrich	Cat#302031
Acetonitrile	Fisher Scientific	Cat#A/0627/17
Ampicillin	Sigma-Aldrich	Cat#A9518
Chloramphenicol	Sigma-Aldrich	Cat#C0378
Streptomycin	Melford	Cat#S0188
L-arabinose	Sigma-Aldrich	Cat#A3256
isopropyl β-D-1-thiogalactopyranoside (IPTG)	Melford	Cat#156000
Tris	VMR Chemicals	Cat#10317P
Sodium di-basic phosphate Na_2HPO_4	Sigma-Aldrich	Cat#S5136

(*Continued on next page*)

Continued

REAGENT or RESOURCE	SOURCE	IDENTIFIER
Tris(2-carboxyethyl)phosphine hydrochloride (TCEP)	Melford	Cat#I236500
Imidazole	Sigma-Aldrich	Cat#I2399
Magnesium Chloride ($MgCl_2$)	Sigma-Aldrich	Cat#M8266
Zinc Chloride ($ZnCl_2$)	VMR Chemicals	Cat#29156
Adenosine Triphosphate (ATP)	Sigma-Aldrich	Cat#A7699
HEPES	Formedium	Cat#HEPES10
Glycine	VMR Chemicals	Cat#10991U
Methanol	Fisher Scientific	Cat#67-56-1
Ammonium Persulfate	BDH	Cat#100323W
TEMED	VWR chemicals	Cat# 443083G
30% Bis Acrylamide	Geneflow	Cat# A2-0072
Fugene 6	Promega	Cat# E2691
MG132	Merck Biosciences	Cat# 474790
Bafilomycin A1	Enzo Life Sciences	Cat#BML-CM110-0100
Opti-MEM	Gibco	Cat#31985
Experimental Models: Cell Lines		
HEK293T	ATCC	RRID:CVCL_0063
Experimental Models: Organisms/Strain		
pET15-Ubiquitin-6His-TAG6	MRC Reagents and Services University of Dundee	DU29174
pET15-Ubiquitin-6His-TAG9	MRC Reagents and Services University of Dundee	DU29175
pET15-Ubiquitin-6His-TAG11	MRC Reagents and Services University of Dundee	DU29176
pET15-Ubiquitin-6His-TAG13	MRC Reagents and Services University of Dundee	DU29177
pET15-Ubiquitin-6His-TAG14	MRC Reagents and Services University of Dundee	DU29185
pET15-Ubiquitin-6His-TAG31	MRC Reagents and Services University of Dundee	DU29178
pET15-Ubiquitin-6His-TAG32	MRC Reagents and Services University of Dundee	DU29179
pET15-Ubiquitin-6His-TAG34	MRC Reagents and Services University of Dundee	DU29180
pET15-Ubiquitin-6His-TAG40	MRC Reagents and Services University of Dundee	DU29181
pET15-Ubiquitin-6His-TAG64	MRC Reagents and Services University of Dundee	DU29182
pET15-Ubiquitin-6His-TAG72	MRC Reagents and Services University of Dundee	DU29183
pET15b 6His UBCH5C S22R C85K	MRC Reagents and Services University of Dundee	DU29199
pET15b 6His UBCH5C S22R F62A C85K	MRC Reagents and Services University of Dundee	DU29756
pGEX6P-1-Cbl	MRC Reagents and Services University of Dundee *	DU12029
pGEX6P-1-Cbl Y371F	MRC Reagents and Services University of Dundee	DU65192
RNF4 (WT)	Tatham et al., 2008	https://www.ncbi.nlm.nih.gov/pubmed/18408734
RNF4-RING	Plechanová et al., 2011	https://www.ncbi.nlm.nih.gov/pubmed/21857666
RNF4x-RING	Plechanová et al., 2011	https://www.ncbi.nlm.nih.gov/pubmed/21857666

(*Continued on next page*)

Continued		
REAGENT or RESOURCE	SOURCE	IDENTIFIER
RNF4x-RINGx	Plechanová et al., 2011	https://www.ncbi.nlm.nih.gov/pubmed/21857666
SUMOx4	Tatham et al, 2008	https://www.ncbi.nlm.nih.gov/pubmed/18408734
Oligonucleotides		
F Mut TAG6 (agaTATACATATGCAGATCTTCG TGtAGACCCTGACTGGTAAGACCATCA C) R mut TAG6GTGATGGTCTTACCAGTCAGGGT CTaCACGAAGATCTGCATATGTATAtc t)	This Manuscript	N/A
See Table S1		N/A
Software, Algorithms and Data Availability		
MS Chemstation software	Agilent Technologies	N/A
Adobe Illustrator	Adobe System, Inc.	https://www.adobe.com/uk/products/illustrator.html?sdid=88X75SKR&mv=search&ef_id=EAIaIQobChMIxu_l2Zih5AIVibHtCh2ZqQcmEAAYASAAEgKKUfD_BwE:G:s&s_kwcid=AL!3085!3!340697722066!e!!g!!adobe%20illustrator&gclid=EAIaIQobChMIxu_l2Zih5AIVibHtCh2ZqQcmEAAYASAAEgKKUfD_BwE
ChemDraw	PerkinElmer	https://www.perkinelmer.com/category/chemdraw?utm_source=Google&utm_medium=cpc&utm_campaign=TEC-DG-GLO-INF-PPC-ZZ-GAW&sfdc_id=7013A000001yj2V&LS=PPC&gclid=EAIaIQobChMIweOr7pih5AIVF-DtCh0jAA_WEAAYASAAEgJ6a_D_BwE
PyMOL	PyMOL.org	https://pymol.org/2/
Prism	GraphPad	N/A
Raw mass spectrometry data has been deposited	https://data.mendeley.com	http://dx.doi.org/10.17632/vv8spnwgyr.1
Other		
4-12% NuPAGE Novex Bris- tris Mini-gels	ThermoFisher Scientific	NP0321BOX
Amicon Ultra-15 3 kDa MWCO centrifugal filter device	Merck	Cat#C7715
HiLoad Superdex-75 16/60 column	GE Life Sciences	Cat#28989333
PD-10 column	GE Life Sciences	Cat#17085101
UV lamp (BLE-8T365)	Spectroline	Cat# ENF280-C
Phos-Tag^{TM}	MRC Reagents and Services University of Dundee	https://mrcintranet.lifesci.dundee.ac.uk/mrc-reagents
L-glutamine	Invitrogen	Cat#25030024
Penicillin-streptomycin	Invitrogen	Cat#15140122
Dulbecco's Modification of Eagle's Medium (DMEM)	Invitrogen	Cat#11960-085
Fetal bovine serum (FBS)	Sigma Aldrich	Cat#F7524
SOC Media	MRC Reagents and Services University of Dundee	N/A
Luria-Bertani (LB)	MRC Reagents and Services University of Dundee	N/A
24-Well Plate	Hampton Research	Cat#HR3-158
Nitrocellulose membrane	GE Life Sciences	Cat#1060002

LEAD CONTACT AND MATERIALS AVAILABILITY

Further information and requests for resources and reagents should be directed to and will be fulfilled by the Lead Contact, Dr. Satpal Virdee (s.s.virdee@dundee.ac.uk). Plasmids generated in this study have been deposited to MRC Reagents and Services with the unique DU number specified in the Key Resources Table (http://mrcppureagents.dundee.ac.uk). All unique/stable reagents generated in this study are available from the Lead Contact with a completed Materials Transfer Agreement.

EXPERIMENTAL MODEL AND SUBJECT DETAILS

H293T cells (donor sex: female) were obtained from ATCC. 293T is a human cell line, derived from the HEK293 cell line, that expresses a mutant version of the SV40 large T antigen (RRID:CVCL_0063). Cells were cultured at 37°C in a humidified incubator under a 5 % CO_2 atmosphere. Dulbecco's modified Eagle medium was used and supplemented with fetal bovine serum and L-glutamine.

Escherichia coli BL21(DE3) or BL21 Rosetta™ (DE3) cells used for protein expression in this study were grown in LB media supplemented with 100 μg mL^{-1} of ampicillin and 34 μg mL^{-1} chloramphenicol (for details see STAR Methods - Expression of Recombinant Proteins).

METHODS DETAILS

Site-Specific Incorporation of *p*Bpa Unnatural Amino Acid into Ubiquitin

pEvol-Bpa plasmid was derived from pEVOL-pBoF (kindly provided by P. Schultz, The Scripps Research Institute). Mutations for incorporation of Bpa were introduced into both copies of *Mj*YRS gene to make the plasmid pEVOL-Bpa (Young et al., 2010; Chin et al., 2002). BL21 cells (50 μL) were co-transformed with the pET-Ubiquitin-6His-TAGx (where x is the Bpa incorporation site) and pEvol-Bpa plasmids using heat shock and recovered in 200 μL SOC media at 37°C for 1 hour and used to inoculate 50 mL Luria-Bertani (LB) containing 100 μg mL^{-1} ampicillin and 34 μg mL^{-1} chloramphenicol. 10 mL overnight culture was then used to inoculate 1 L LB broth containing the same concentrations of antibiotics. The cells were grown until OD_{600} reached ~0.6 and the culture was divided into two 500 mL portions. One portion was supplemented with 1 mM *p*-Benzoyl-L-phenylalanine (Bpa; Bachem) and the other served as a control where Bpa was withheld. The cultures were incubated for 20 mins (37°C, 200 rpm), or until the OD_{600} reached 0.6-0.7, and protein expression was induced by adding 0.02 % arabinose and 1 mM isopropyl β-D-1-thiogalactopyranoside (IPTG). The cultures were incubated for 5 hours (37°C, 200 rpm). The cells were harvested and suspended in 10 mL BugBuster® Protein Extraction (Merk Millipore) reagent before transferring to 50 mL falcon tube. The lysates were incubated for 20 minutes and then clarified by centrifugation before transferring to 50 mL falcon tube containing 1 mL Ni-NTA agarose beads and incubated for 1 hour with gentle shaking. The resin was centrifuged (4°C, 1000 rpm) and washed with wash buffer (20 mM Na_2HPO_4, pH 7.5, 25 mM imidazole). Finally, the protein was eluted with 200 μL elution buffer (20 mM Na_2HPO_4, pH 7.5, 300 mM imidazole). A 20 μL aliquot from the elution fraction was mixed with equal amount of 4X SDS loading buffer and loaded onto 4-12% SDS-PAGE gel. The proteins were separated at 200 V using MES buffer for 30 minutes and detected using Coomassie blue staining. A separate 20 μL protein was analyzed by LC-MS. LC-MS was carried out with an Agilent 1200 LC-MS system fitted with a Max-Light Cartridge flow cell coupled to a 6130 Quadrupole spectrometer. An Agilent ZORBAX 300SB-C3 5 μm, 2.1 x 150 mm column was employed unless otherwise stated. The solvent system consisted of 0.05 % trifluoroacetic acid in H_2O as buffer A, and 0.04 % TFA acid in acetonitrile as buffer B. Protein UV absorbance was monitored at 214 and 280 nm. MS acquisition was carried out in positive ion mode and total protein masses were calculated by deconvolution within the MS Chemstation software (Agilent Technologies).

Fractions containing the *p*Bpa incorporated-Ub were pooled concentrated with an Amicon Ultra-15 3 kDa MWCO centrifugal filter device (Millipore). The sample was desalted into 10 mM Tris-HCl pH 7.5 using a PD-10 column (GE Life Sciences). DTT (1 mM) was added to the sample, followed by hexahistidine tag cleavage with UCH-L3 (Virdee et al., 2010), at a final concentration of 15 μg mL^{-1}. The sample was incubated at 37°C for 2 hours to remove the N-terminal His tag. Bpa incorporated-Ub was further purified by semi-preparative HPLC and the fractions were lyophilized yielding approximately 4-6 mg of Ub-*p*Bpa.

Expression of UBE2D3(S22R/C85K) Recombinant Protein

S22R and C85K were introduced into UBE2D3 by using site-directed mutagenesis. The cells were grown until OD_{600} reached 0.6-0.7 at 37°C, 200 rpm. Once OD_{600} reached 0.6~0.7, protein expression was induced by adding IPTG (1 mM) and incubated at 37°C for 3 h. The cells were harvested and resuspended in buffer (20 mM Na_2HPO_4, pH 7.5, 150 mM NaCl, 1 mM TCEP, complete protease inhibitor cocktail (EDTA-free, Roche). Lysozyme was added (0.5 mg mL^{-1}) and cells were incubated on ice for 30 min followed by sonication. Clarified lysates containing His6-tagged UBE2D3(S22R/C85K) were loaded onto Ni-NTA resin and washed with buffer (20 mM pH 7.5, Na_2HPO_4, pH 7.5, 25 mM imidazole, 150 mM NaCl, 1 mM TCEP), followed by elution with elution buffer (20 mM pH 7.5, Na_2HPO_4, pH 7.5, 300 mM imidazole, 150 mM NaCl, 1 mM TCEP). Samples were further purified by size-exclusion chromatography with a HiLoad Superdex-75 16/60 column (GE Healthcare) with running buffer (20 mM Na_2HPO_4, pH 7.5, 150 mM NaCl, 1 mM TCEP).

Preparation of Biotin-UbBpa31

Lyophilized UbBpa31 with an N-terminal MGCSSG cysteine-containing motif (10 mg) was reconstituted in 1 mL 10% DMSO/90% 0.5 mM TCEP (aq) and incubated at 23°C for 45 mins with gentle mixing, followed by the addition of 5 molar equivalents of EZ-link iodo-acetyl PEG2-Biotin (Thermofisher) in reaction buffer (50 mM Na_2HPO_4, 150 mM NaCl, 0.5 mM TCEP). The reaction was incubated at 23°C with gentle shaking for 1 h and monitored to completion by LC-MS. Product was then purified by preparative HPLC at a flow rate and lyophilized yielding biotin-UbBpa31 (6-8 mg).

Preparation of Isopeptide-linked photoABPs

To generate the photoABP-Bpa probes UBE2D3(S22R C85K) (200 μM) was incubated with UbBpa (200 μM) and His_6–Uba1 (1 μM) at 35°C for 26 h conjugation buffer (50 mM Tris, pH 10.0, 150 mM NaCl, 3 mM ATP, 5 mM $MgCl_2$, 1 mM TCEP). The E2– UbBpa conjugate was applied onto a HiLoad 16/60 Superdex 75 gel filtration column (GE Healthcare) (20 mM HEPES, pH 7.5, 150 mM NaCl, 1 mM TCEP). The purified photoABP-Bpa probes were concentrated to 2 mg ml^{-1}, and stored at -80°C. Biotin-photoABP-Bpa31 probe was prepared using the same procedure.

Expression of Recombinant RNF4 Protein

Cloning, expression and purification of linear fusion of two RNF4 RING domains, and associated mutants, has been described previously (Plechanovová et al., 2011). The fusion of two RING domain of RNF4 were expressed in *E. coli* Rosetta (DE3) cells (Novagen). The cells were grown until OD_{600} reached 0.6∼0.7 at 37°C, 200 rpm. Once the OD_{600} reached 0.6-0.7, the protein expression was induced by adding IPTG (1 mM) and incubated overnight at 16°C, 200 rpm.

The cells were harvested and resuspended in lysis buffer (50 mM Tris, pH 7.5, 0.5 M NaCl, 10 mM imidazole, 2 mM benzamidine, complete protease inhibitor cocktail (EDTA-free, Roche)) and cells were lysed by sonication. His6-MBP-fusion proteins were purified by Ni-NTA (Qiagen) chromatography, followed by cleavage with TEV protease at 4°C overnight. To remove any uncleaved fusion protein, His6-tagged MBP, as well as His6-tagged TEV protease, material was depleted against fresh Ni-NTA resin followed by size-exclusion chromatography with a HiLoad Superdex 75 16/60 column (GE Healthcare) (20 mM Tris, 150 mM NaCl, 1 mM TCEP, pH 7.5).

Expression of c-Cbl and c-Cbl (Y371F) Recombinant Protein

BL21(DE3) cells (50 μL) were transformed with the pGEX6P-1-Cbl plasmid and recovered in 200 μL SOC media at 37°C for 1 hour and used to inoculate 50 mL Luria-Bertani (LB) containing 100 μg mL^{-1} ampicillin. 10 mL overnight culture was then used to inoculate LB broth containing the same concentration of antibiotic and 0.2 mM zinc chloride. The cells were grown until OD_{600} reached 0.6-0.7 at 37°C, 200 rpm. Once the OD_{600} reached 0.6∼0.7, protein expression was induced by adding 1 mM IPTG and left overnight at 16°C, 200 rpm. The cells were harvested and resuspended in buffer (50 mM Hepes, pH 7.5, 0.5 M NaCl, 1 mM TCEP) and lysed by sonication. The lysates were incubated with glutathione sepharose beads for 1 hour with gentle shaking. The resin was centrifuged (4°C, 1000 rpm) and washed with buffer (50 mM HEPES, pH 7.5, 150 mM NaCl, 1 mM TCEP), followed by cleavage with Rhinovirus 3C protease at 4°C overnight. Cleaved protein was further purified by size-exclusion chromatography with a HiLoad Superdex 200 16/600 column (GE Healthcare) (20 mM HEPES, 150 mM NaCl, 1 mM TCEP, pH 7.5).

c-Cbl Phosphorylation

Purified c-Cbl (3 μM) was phosphorylated by incubating with Src kinase (1.5 μM), 10 mM $MgCl_2$, 5 mM ATP at 37°C, 45 mins. Samples (15 μl) were collected and mixed well with 4X LDS loading buffer (ThermoFisher), followed by boiling before loading onto 7.5 % acrylamide phos-tag gel. The proteins were separated at 160 V using MOPS buffer for 60 mins and analysed using Coomassie staining and western blot.

Furthermore, ATP-dependent phosphorylation and photo-cross linking of c-Cbl with photoABP-Bpa31 (5 μM) was analysed using Coomassie staining. Samples (15 μl) were collected and mixed well with 4X LDS loading buffer, followed by boiling them for 5 mins at 95°C before loading onto 4-12% SDS-PAGE gel using MOPS running buffer and analysed using Coomassie staining. Moreover, gels were blotted and analysed using western blot with anti-Cbl (1:5000 dilution) as primary and anti-mouse (1:10000 dilution) as secondary antibodies.

UV Irradiation Conditions for Photo-Cross-Linking

Photo-cross linking reactions (45 μL) were performed in a 24-well plate (Cryshem HR3-158, Hampton Research) in reaction buffer (20 mM HEPES, pH 7.5, 150 mM NaCl, 1 mM TCEP). Samples were divided into two portions. One portion was irradiated at 365 nm on ice at a distance of 2 cm from a handled UV lamp (BLE-8T365, Spectroline), for 10-30 min and the other portion was preserved in the dark. For purified proteins such as RNF4-RING (5-10 μM), c-Cbl (3 μM) and c-Cbl Y371F (3 μM), photo-cross linking reactions were performed with photoABP-Bpa31 probe (5-40 μM) and irradiated with UV. Samples were resolved by SDS-PAGE and visualized by Coomassie staining or immunoblotting. Control experiments were performed under the same conditions.

Photo-Crosslinking in Cell Extracts

HEK293 cells were transfected with plasmids expressing GFP-Cbl, GST-Src and GFP-Cbl. The cells were lysed in lysis buffer (50 mM Na_2HPO_4, 10 mM Glycerophosphate, 50 mM Sodium Fluoride, 5 mM Sodium Pyrophosphate, 1 mM Sodium Vanadate, 0.25 M Sucrose, 50 mM NaCl, 0.2 mM PMSF, 1 mM Benzamidine, 10 μM TCEP, 1 % NP-40). Probe photoABP-Bpa31 (5-10 μM) was mixed with cell lysate and UV irradiated (10 mins) using the photocrosslinking procedure described in the general method. Samples were analysed by 4-12 % SDS-PAGE gel using MOPS running buffer (160 V, 60 mins) and visualized by immunoblotting with anti-Cbl (1:5000 dilution) as primary and anti-mouse (1:10000 dilution) as secondary antibodies.

Phos-tag™ Gel Electrophoresis

To assess Src-mediated c-Cbl phosphorylation, we poured resolving gels (7.5 % acrylamide/bis-acrylamide, 375 mM Tris-HCl pH 8.8, 0.1% sodium dodecyl sulfate (SDS), 100 μM $MnCl_2$, 50 μM Phos-tag™, 0.05 % (w/v) ammonium persulphate (APS),

0.0625 % (v/v) tetramethylethylenediamine (TEMED)) and stacking gels (4 % acrylamide/bis-acrylamide, 125 mM Tris-HCl pH 6.6, 0.1 % SDS, 0.05 % (w/v) APS, 0.1 % (v/v) TEMED), degassing with argon, then allowing polymerization at room temperature for three hours. Cell extracts (50 μg) were boiled in LDS-sample buffer and supplemented with 10 mM $MnCl_2$ before loading. Electrophoresis was performed at 70 V through the stacking gel and 130 V through the resolving gel using running buffer (25 mM Tris-HCl, 192 mM Glycine, 0.1% SDS), before staining with Coomassie dye, or washing 3 x 20 min in transfer buffer (48 mM Tris-HCl, 39 mM glycine, 20% methanol) supplemented with 10 mM EDTA and 0.05 % SDS to chelate manganese, followed by 1 x 20 min in transfer buffer supplemented with 0.05 % SDS. Protein was then transferred to 0.45 μm nitrocellulose membrane in transfer buffer at 100 V, 3 hr, 4°C.

Cell Culture, Transfection and Lysis

293T cells were cultured (37°C, 5 % CO_2) in Dulbecco's modified Eagle's medium (DMEM) supplemented with 10 % (v/v) fetal bovine serum (FBS), 2.0 mM L-glutamine and antibiotics (100 units mL^{-1} penicillin, 0.1 mg mL^{-1} streptomycin). Cells were seeded at a density of 4 x 10^6 in 100 mm dishes. 18 hr post seeding, cell transfections (2 μg DNA, empty vector (pcDNA (Thermo Fisher)), pcDNA and GST-Src, pcDNA and GFP-c-Cbl, or GST-Src and GFP-c-Cbl) were performed using 5 μL Fugene-6 (Promega) in 200 μL Eagle's Minimum Essential Medium (Opti-MEM). MG132 (25 μM) was added to cells 90 min before harvesting. Cells were rinsed and collected with ice-cold PBS, and extracted with ice-cold lysis buffer (50 mM Tris-HCl pH 7.5, 10 mM sodium 2-glycerophosphate, 50 mM sodium fluoride, 5.0 mM sodium pyrophosphate, 1.0 mM sodium orthovanadate, 0.27 M sucrose, 50 mM NaCl, 0.2 mM phenylmethanesulfonyl fluoride (PMSF), 1.0 mM benzamidine, 10 μM TCEP, 1% NP-40) on ice for 30 min. Lysates were clarified by centrifugation at 4°C for 20 min at 21,100g. Supernatants were collected and protein concentration was determined by Bradford assay.

Activity-Based Proteomic Profiling of EGF-Stimulated HEK293 Cells

293T cells were seeded in 150 mm dishes at a density of 5 x 10^6 and cultured (37°C, 5 % CO_2) in Dulbecco's modified Eagle's medium (DMEM) supplemented with 10 % (v/v) fetal bovine serum (FBS), 2.0 mM L-glutamine and antibiotics (100 units mL^{-1} penicillin, 0.1 $mgmL^{-1}$ streptomycin). The next day, media was replaced for DMEM lacking FBS. The following day, cells were treated with 20 μM MG132 and 200 nM Bafilomycin for 6 hours at 37°C, then with or without recombinant EGF 100 $ngmL^{-1}$ (Thermo Fisher Scientific, PHG0311) for 15 minutes at 37°C. Dishes were transferred on to ice, washed, resuspended in ice-cold PBS, and washed twice at 4°C, and lysates extracted in ice-cold lysis buffer. 293T cells were treated with biotinylated probe (biotin-photoABP-Bpa31) (10 μM). Samples were divided and irradiated with UV for 1 hour or UV was withheld. Biotin enrichment was then carried out against streptavidin resin followed by on-resin tryptic digestion and LC-MS/MS analysis and data processing, as previously described, with an exception being hardware parametrization with an inclusion list of theoretical Cbl tryptic peptides (Pao et al., 2018).

QUANTIFICATION AND STATISTICAL ANALYSIS

Data were presented as mean ± standard error determined from technical replicate. Statistical analysis was performed with GraphPad Prism (version 6.0). Figures 4E, S4H, and S4I were filtered against the PFAM domain terms "RING, HECT, IBR, zf-UBR and DUBs". All biological experiments were performed at least twice.

DATA AND CODE AVAILBILITY

The mass spectrometry raw data files have been deposited in the Mendeley database (https://dx.doi.org/10.17632/vv8spnwgyr.1).

Cell Chemical Biology, Volume *27*

Supplemental Information

Photocrosslinking Activity-Based Probes for Ubiquitin RING E3 Ligases

Sunil Mathur, Adam J. Fletcher, Emma Branigan, Ronald T. Hay, and Satpal Virdee

Photocrosslinking Activity-Based Probes for Ubiquitin RING E3 Ligases

Supplemental Information

Sunil Mathur[a], Adam J. Fletcher[a], Emma Branigan[b], Ronald T. Hay[b] and Satpal Virdee*[a]

[a] Dr. S. Mathur, Dr. A.J. Fletcher, Dr S. Virdee
MRC Protein Phosphorylation and Ubiquitylation Unit
University of Dundee
Scotland, UK
E-mail: s.s.virdee@dundee.ac.uk

[b] Dr. E. Branigan, Prof. R.T. Hay
Division of Gene Regulation and Expression
University of Dundee
Scotland, UK

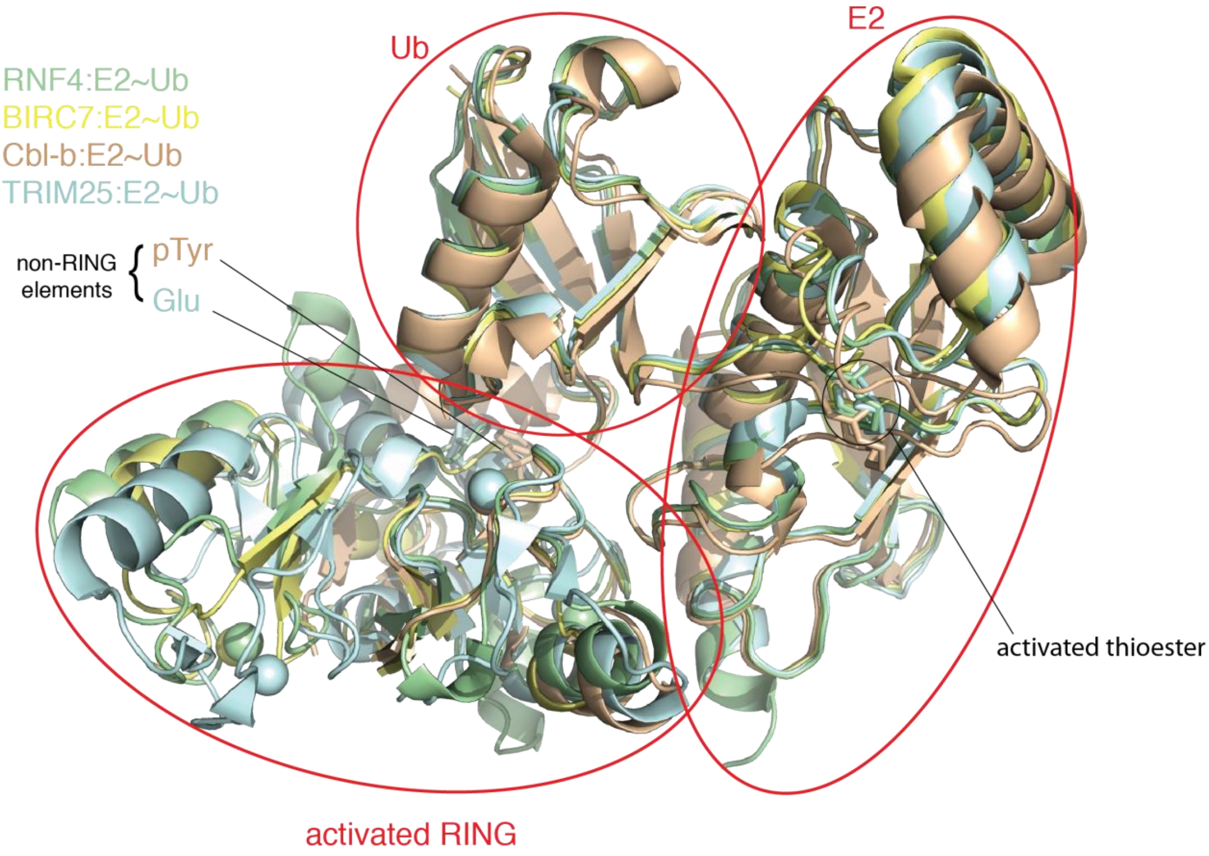

Figure S1. Structural superposition of activated RING:E2~Ub complexes, related to Figure 1. Activated RING E3s bind E2~Ub and induce a "closed" E2~Ub conformation which activates the thioester for aminolysis (NB, in the presented crystal structures the labile thioester has been replaced with an ester or isopeptide linkage by mutation of the E2 catalytic cysteine to serine or lysine, respectively). The closed conformation is induced by binding of the RING to a composite E2~Ub interface. Of particular significance, the Ub component is held in the closed conformation by interactions with RING protomer 2 in the case of RNF4 and BIRC7. For Cbl-b, a phosphotyrosine moiety (pTyr) serves as a non-RING element and facilitates the closed conformation. In the case of TRIM25, dimerization and the presence of a non-RING glutamate (Glu) residue facilitates the closed conformation.

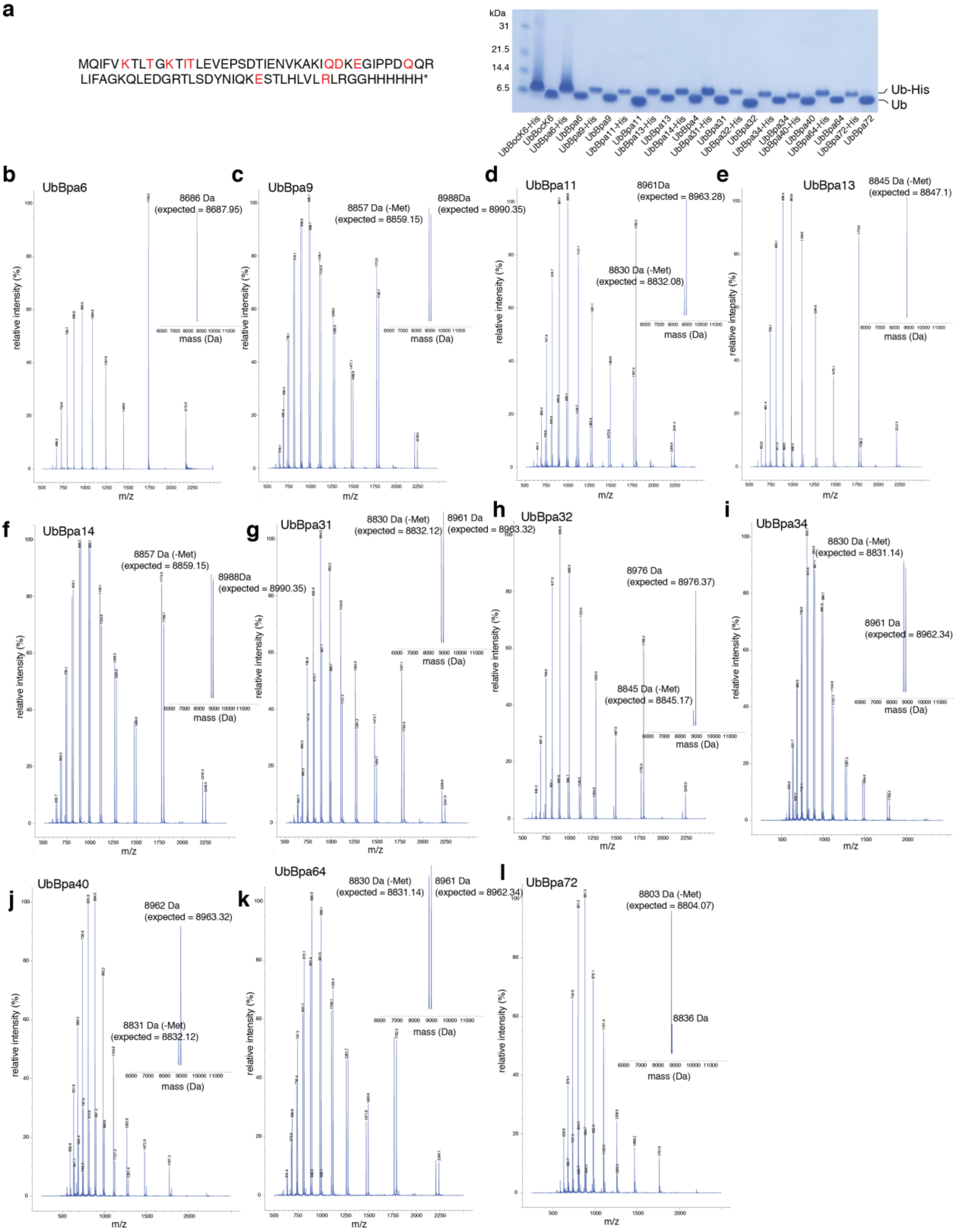

Figure S2. Incorporation of the photocrosslinking amino acid p-benzoyl-L-phenylalanine (Bpa) into RING-proximal sites of ubiquitin, related to Figure 2. a) Ubiquitin sequence with mutated residues highlighted in red and SDS-PAGE analysis and visualization by Coomasie staining. As a positive control, the reference amino acid t-butyloxycarbonyl-L-lysine (BocK) was incorporated into Ub at position 6 (Pao et al., 2016). To facilitate purification a C-terminal His tag was appended to Ub which was subsequently removed by treatment with the deubiquitinating enzyme UCH-L3 (Virdee et al., 2010). For each mutant, samples pre- and post-UCH-L3 treatment were analysed. **b)** Electrospray

ionization mass spectra for ubiquitin Bpa mutants. Spectra correspond to Ub after cleavage of the C-terminal His-tag. With the exception of the amber mutant clone for position 6, Ub is expressed with an N-terminal MGS motif. Introduction of the DNA coding sequence for this facilitated cloning. For these latter clones the N-terminal methionine is cleaved by cellular methionyl aminopeptidase to various degrees. UbBpa6, observed mass = 8686 Da; expected = 8787.95 Da. **c)** UbBpa9 (-Met), observed mass = 8857 Da; expected = 8859.15 Da. UbBpa9, observed mass = 8988 Da; expected = 8990.35 Da. **d)** UbBpa11 (-Met), observed mass = 8830 Da; expected = 8832.08 Da. UbBpa11, observed mass = 8961 Da; expected = 8963.28 Da. **e)** UbBpa13 (-Met), observed mass = 8845 Da; expected = 8847.1 Da. **f)** UbBpa14 (-Met), observed mass = 8857 Da; expected = 8859.15 Da. UbBpa14, observed mass = 8988 Da; expected = 8990.35 Da. **g)** UbBpa31 (-Met), observed mass = 8830 Da; expected = 8832.12 Da. UbBpa31, observed mass = 8961 Da; expected = 8963.32 Da. **h)** UbBpa32 (-Met), observed mass = 8845 Da; expected = 8845.17 Da. UbBpa32, observed mass = 8976 Da; expected = 8976.37 Da. **i)** UbBpa34 (-Met), observed mass = 8830 Da; expected = 8831.14 Da. UbBpa34, observed mass = 8961 Da; expected = 8962.34 Da. **j)** UbBpa40 (-Met), observed mass = 8831 Da; expected = 8832.12 Da. UbBpa40, observed mass = 8962 Da; expected = 8963.32 Da. **k)** UbBpa64 (-Met), observed mass = 8830 Da; expected = 8831.14 Da. UbBpa64, observed mass = 8961 Da; expected = 8962.34 Da. **l)** UbBpa72 (-Met), observed mass = 8803 Da; expected = 8804.07 Da. Observed peak at 8836 Da corresponds to an unidentified adduct.

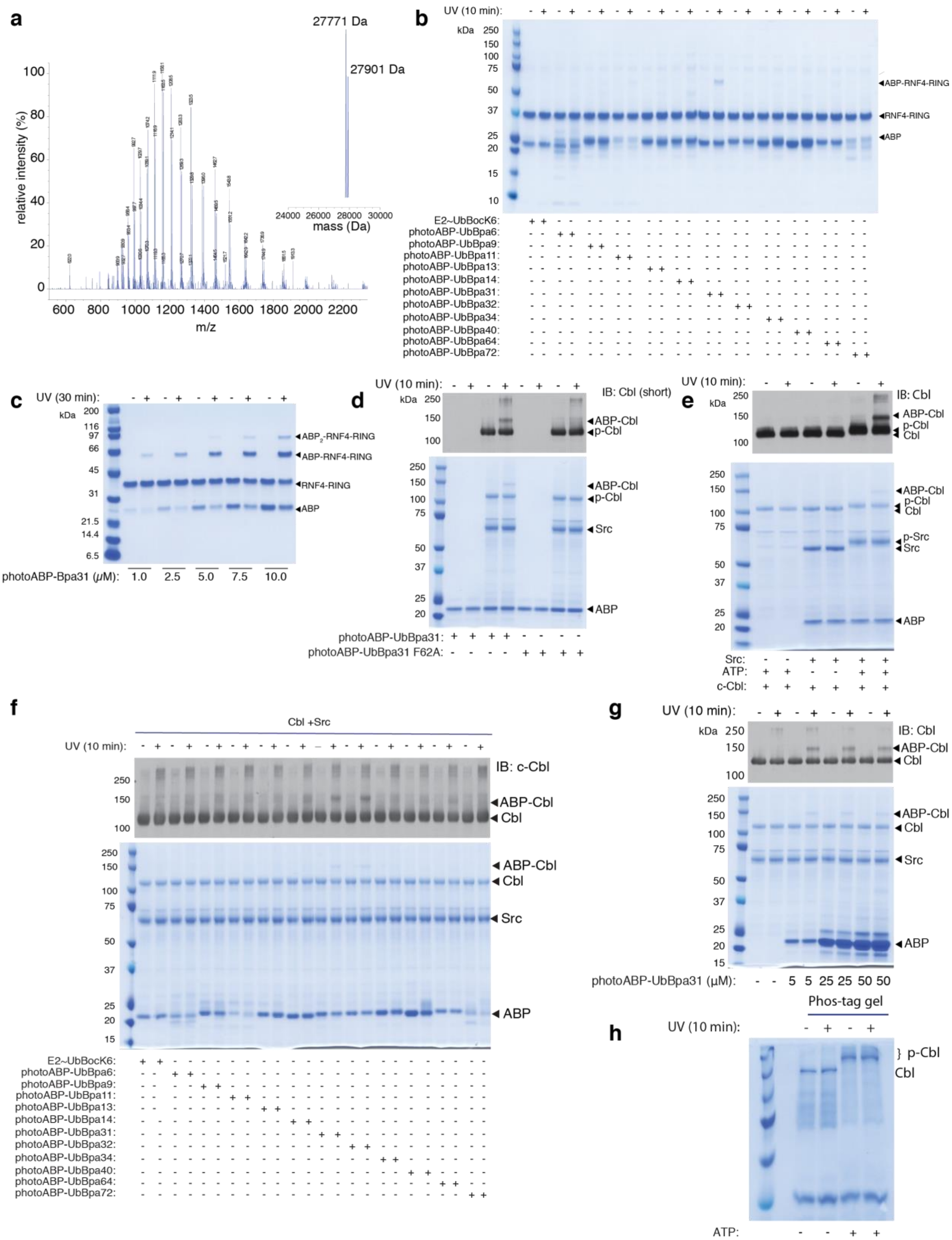

Figure S3. Supplemental photoABP characterization data, related to Figure 3 and 4. a) Raw ESI-MS spectrum for sample for photoABP-Bpa31. photoABP Bpa31 (-Met), observed mass = 27769 Da; expected mass = 27777.67 Da. photoABP-UbBpa31 (-Met), observed mass = 27901 Da; expected mass = 27908.87 Da. **b)** Assessment of RNF4-RING photocrosslinking efficiency for incorporation of Bpa at 10 different Ub positions within isopeptide-linked E2~Ub conjugate. Significant incorporation was only achieved with incorporation of Bpa at position 31, furnishing

probe photoABP-UbBpa31. The reference amino acid *t*-butyloxycarbonyl-L-lysine (BocK) and Bpa were incorporated at position 6 as this site is highly permissive to unnatural amino acid incorporation and served as positive controls (Virdee et al., 2010). **c)** Dose responsive photocrosslinking of RNF4-RING. Photocrosslinking efficiency of RNF4-RING was responsive to increasing concentrations of photoABP-UbBpa31. **d)** Photocrosslinking of phosphorylated c-Cbl with photoABP-UbBpa31 and the photoABP-UbBpa31 F62A control probe. Purified c-Cbl (3 μM) was preincubated with c-Src (1.5 μM) in the presence of ATP (5 mM) for 45 min at 37 ºC. Reaction mixture was then profiled with the specified probes (5 μM). The probe photoABP-Bpa31 undergoes c-Cbl crosslinking whereas photoABP-Bpa31 F62A does not. **e)** Photocrosslinking of Src treated c-Cbl is strictly ATP-dependent. Purified c-Cbl (3 μM) was preincubated with c-Src (1.5 μM) in the presence (5 mM) or absence of ATP for 45 min at 37 °C. Reaction mixture was then profiled with the specified probes (5 μM). The probe photoABP-Bpa31 undergoes c-Cbl crosslinking only in the presence of Src and ATP. **f)** Comparison of photocrosslinking efficiency for Bpa incorporation at multiple positions within ubiquitin. c-Cbl (3 μM) was phosphorylated by incubation with c-Src (1.5 μM) prior to probe analysis. Only incorporation of Bpa at positions 31 and 32 furnishes a functional probe. **g)** Dose-response analysis of Cbl crosslinking. c-Cbl (3 μM) was phosphorylated by incubation with c-Src (1.5 μM) prior to probe analysis. No increase in Cbl labelling efficiency was observed as a result of increasing photoABP-UbBpa31 concentration beyond 5 μM. **h)** Phostag SDS-PAGE analysis of c-Src treated c-Cbl. Reduced electrophoretic mobility of c-Cbl is only observed in the presence of ATP. As the gel shift is quantitative, this indicates that phosphorylation must too be quantitative. However, crosslinking efficiency with photoABP-UbBpa31 was not dose responsive beyond 5 μM. This is consistent with c-Cbl becoming phosphorylated at multiple sites but substoichiometrically at Y371.

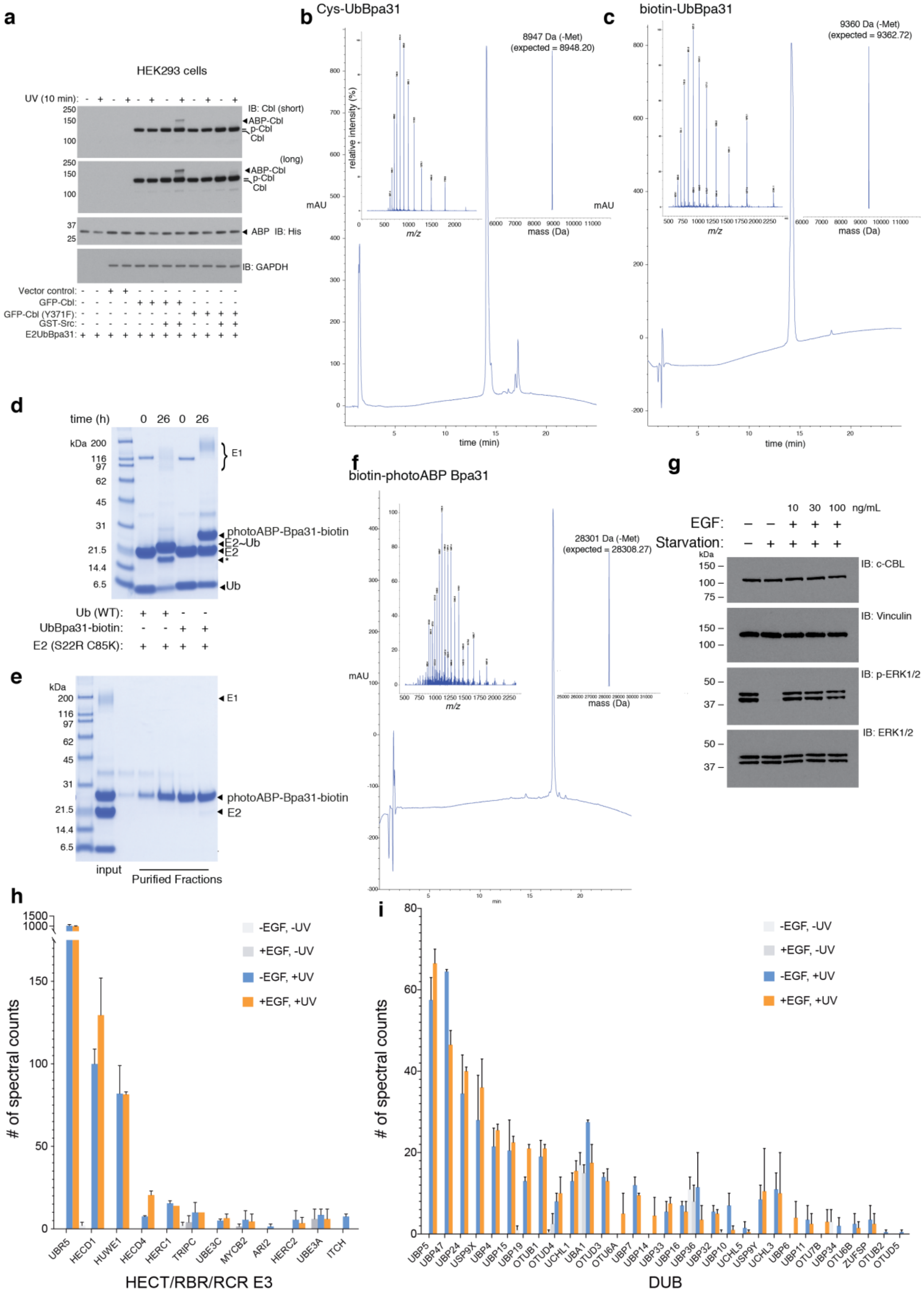

Figure S4. Supplemental blot for Figure 4b and characterization data for construction of biotinylated photoABP-Bpa31, confirmation of receptor tyrosine kinase activation and assessment of non-RING E3 photocrosslinking related to Figure 4. a)

Photocrosslinking of cellular Cbl remains strictly dependent on the presence of Y371. C-Cbl (3 μM) was phosphorylated by incubation with c-Src (1.5 μM) prior to probe analysis. **b)** Biotin labelling of Cys-tagged UbBpa31 and enzymatic conjugation to E2. LC-MS characterization of UbBpa31 expressed with an N-terminal MGCSSG labelling motif (observed mass = 8947 Da; expected mass = 8948.2 Da). **c)** Cysteine labelling motif was alkylated with EZ-Link Iodoacetyl-PEG2-Biotin (Thermofisher). Product was purified by preparative RP-HPLC and characterized by LC-MS (observed mass = 9360 Da; expected mass = 9362.72 Da). **d)** Refolded biotin-tagged UbBpa31 was enzymatically conjugated onto E2. **e)** The probe was purified by size-exclusion chromatography. **f)** LC-MS analysis for biotin-photoABP-Bpa31. HPLC chromatogram measured at 214 nm. photoABPBpa31 (-Met), observed mass = 28301 Da; expected mass = 28308.27 Da. **g)** EGF-dependent receptor activation is confirmed by immunoblotting for ERK1/2 phosphorylation. HEK293T cells were serum-starved and stimulated with recombinant EGF. Cells were treated with the proteasome inhibitor MG132 prior to stimulation. N.B. for proteomic experiment cells were treated with MG132 and bafilomycin. **h**) Detection of other ubiquitin system components by activity-based proteomics with biotinylated photoABP-UbBpa31. Spectral counts obtained from ABP-profiled HEK293T cells. Search results were filtered against the PFAM domain term “HECT, IBR and zf-UBR” and only RING E3s with >2 spectral counts in any replicate experiment were plotted. Cells were serum-starved and either treated with or without EGF and with or without UV irradiation. Errors bar correspond to the standard error from two technical replicates. **i**) As above but DUBs were filtered using a combination of PFAM domain terms and manual curation.

REAGENT or RESOURCE	SOURCE	IDENTIFIER
Oligonucleotides		
F Mut TAG9 (GATCTTCGTGAAGACCCTGtagGGTAAGACCATCACTCTCG)<br>R mut TAG9 (CGAGAGTGATGGTCTTACCctaCAGGGTCTTCACGAAGATC)	This Manuscript	N/A
F Mut TAG11 (CGTGAAGACCCTGACTGGTtagACCATCACTCTCGAAGT)<br>R mut TAG11 (ACTTCGAGAGTGATGGTctaACCAGTCAGGGTCTTCACG)	This Manuscript	N/A
F Mut TAG13 (GACCCTGACTGGTAAGACCtagACTCTCGAAGTGGAGCCGA)<br>R mut TAG13 (TCGGCTCCACTTCGAGAGTctaGGTCTTACCAGTCAGGGTC)	This Manuscript	N/A
F Mut TAG14 (GACCCTGACTGGTAAGACCATCtagCTCGAAGTGGAGCCGAGTGACA)<br>R mut TAG14 (TGTCACTCGGCTCCACTTCGAGctaGATGGTCTTACCAGTCAGGGTC)	This Manuscript	N/A
F Mut TAG31 (GAGAATGTCAAGGCAAAGATCtagGACAAGGAAGGCATCCCTCCT)<br>R mut TAG31 (AGGAGGGATGCCTTCCTTGTCctaGATCTTTGCCTTGACATTCTC)	This Manuscript	N/A
F Mut TAG32 (GTCAAGGCAAAGATCCAAtagAAGGAAGGCATCCCTCCT)<br>R mut TAG32 (AGGAGGGATGCCTTCCTTctaTTGGATCTTTGCCTTGAC)	This Manuscript	N/A
F Mut TAG34 (GCAAAGATCCAAGACAAGtagGGCATCCCTCCTGACCAG)<br>R mut TAG34 (CTGGTCAGGAGGGATGCCctaCTTGTCTTGGATCTTTGC)	This Manuscript	N/A
F Mut TAG40 (GAAGGCATCCCTCCTGACtAGCAGAGGTTGATCTTTG)<br>R mut TAG40 (CAAAGATCAACCTCTGCTaGTCAGGAGGGATGCCTTC)	This Manuscript	N/A
F Mut TAG64 (GACTACAACATCCAGAAAtAGTCCACCCTGCACCTGG)<br>R mut TAG64 (CCAGGTGCAGGGTGGACTaTTTCTGGATGTTGTAGTC<br>)	This Manuscript	N/A

F Mut TAG72<br>(CACCCTGCACCTGGTCCTCtagCTCCGAGGTGGGCATCACC)<br>R mut TAG72<br>(GGTGATGCCCACCTCGGAGctaGAGGACCAGGTGCAGGGTG)	This Manuscript	N/A
UbCH5C -S22R<br>NW2690<br>(GACCCTCCAGCACAATGTcgTGCAGGTCCAGTTGGGGA)<br>NW2691<br>(TCCCCAACTGGACCTGCAcgACATTGTGCTGGAGGGTC)<br>C85K<br>NW4685<br>(TAACAGTAATGGCAGCATTaaaCTCGATATTCTAAGATCAC)<br>NW4686<br>(GTGATCTTAGAATATCGAGtttAATGCTGCCATTACTGTTA)	This Manuscript	N/A
UbCH5C -F62A<br>NW4687<br>(CATTTTCCTACAGACTACCCCgcCAAACCACCTAAGGTTGCATT)<br>NW4688<br>(AATGCAACCTTAGGTGGTTTGgcGGGGTAGTCTGTAGGAAAATG)	This Manuscript	N/A
Cbl<br>NW6441<br>(CAGGAACAATATGAATTATtCTGTGAGATGGGCTCCACA)<br>NW6442<br>(TGTGGAGCCCATCTCACAGaATAATTCATATTGTTCCTG)	This Manuscript	N/A

Table S1. List of oligonucleotides generated in this study. Related to STAR Methods.
